# Terminology and concepts for the characterization of in vivo MR spectroscopy methods and MR spectra: Background and experts' consensus recommendations

**DOI:** 10.1002/nbm.4347

**Published:** 2020-08-17

**Authors:** Roland Kreis, Vincent Boer, In‐Young Choi, Cristina Cudalbu, Robin A. de Graaf, Charles Gasparovic, Arend Heerschap, Martin Krššák, Bernard Lanz, Andrew A. Maudsley, Martin Meyerspeer, Jamie Near, Gülin Öz, Stefan Posse, Johannes Slotboom, Melissa Terpstra, Ivan Tkáč, Martin Wilson, Wolfgang Bogner, Experts' Working Group on Terminology for MR Spectroscopy

**Affiliations:** ^1^ Department of Radiology, Neuroradiology, and Nuclear Medicine and Department of Biomedical Research University Bern Bern Switzerland; ^2^ Danish Research Centre for Magnetic Resonance, Funktions‐ og Billeddiagnostisk Enhed Copenhagen University Hospital Hvidovre Hvidovre Denmark; ^3^ Department of Neurology, Hoglund Brain Imaging Center University of Kansas Medical Center Kansas City Kansas USA; ^4^ Centre d'Imagerie Biomedicale (CIBM) Ecole Polytechnique Fédérale de Lausanne (EPFL) Lausanne Switzerland; ^5^ Department of Radiology and Biomedical Imaging & Department of Biomedical Engineering Yale University New Haven Connecticut USA; ^6^ The Mind Research Network Albuquerque New Mexico USA; ^7^ Department of Radiology and Nuclear Medicine Radboud University Medical Center Nijmegen The Netherlands; ^8^ Division of Endocrinology and Metabolism, Department of Internal Medicine III & High Field MR Centre, Department of Biomedical Imaging and Image guided Therapy Medical University of Vienna Vienna Austria; ^9^ Laboratory of Functional and Metabolic Imaging (LIFMET) Ecole Polytechnique Fédérale de Lausanne Lausanne Switzerland; ^10^ Sir Peter Mansfield Imaging Centre, School of Medicine University of Nottingham Nottingham UK; ^11^ Department of Radiology, Miller School of Medicine University of Miami Miami Florida USA; ^12^ Center for Medical Physics and Biomedical Engineering Medical University of Vienna Vienna Austria; ^13^ High Field MR Center Medical University of Vienna Vienna Austria; ^14^ Douglas Mental Health University Institute and Department of Psychiatry McGill University Montreal Canada; ^15^ Center for Magnetic Resonance Research, Department of Radiology University of Minnesota Minneapolis Minnesota USA; ^16^ Department of Neurology University of New Mexico School of Medicine Albuquerque New Mexico USA; ^17^ Department of Radiology, Neuroradiology, and Nuclear Medicine University Hospital Bern Bern Switzerland; ^18^ Centre for Human Brain Health and School of Psychology University of Birmingham Birmingham UK; ^19^ High Field MR Center, Department of Biomedical Imaging and Image‐guided Therapy Medical University of Vienna Vienna Austria

**Keywords:** MR spectroscopic imaging, MR spectroscopy, spectroscopic quantitation, standardization

## Abstract

With a 40‐year history of use for in vivo studies, the terminology used to describe the methodology and results of magnetic resonance spectroscopy (MRS) has grown substantially and is not consistent in many aspects. Given the platform offered by this special issue on advanced MRS methodology, the authors decided to describe many of the implicated terms, to pinpoint differences in their meanings and to suggest specific uses or definitions. This work covers terms used to describe all aspects of MRS, starting from the description of the MR signal and its theoretical basis to acquisition methods, processing and to quantification procedures, as well as terms involved in describing results, for example, those used with regard to aspects of quality, reproducibility or indications of error. The descriptions of the meanings of such terms emerge from the descriptions of the basic concepts involved in MRS methods and examinations. This paper also includes specific suggestions for future use of terms where multiple conventions have emerged or coexisted in the past.

## Graphical abstract

This work defines terms and explains concepts used to describe all aspects of MR spectroscopy, starting from the definition of the MR signal and its theoretical basis to acquisition methods, processing and quantification procedures, as well as terms involved in describing results, including quality aspects, reproducibility or indications of error. Also featured are specific suggestions for future use of terms where multiple conventions have emerged or previously coexisted.

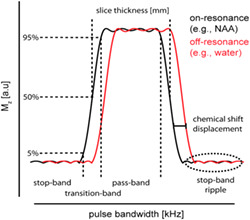

## 1 INTRODUCTION

With its roots in high resolution nuclear magnetic resonance (NMR) and a 40‐year history of use for in vivo studies, the terminology used to describe in vivo MR spectroscopy (MRS) * has grown substantially, with some inconsistent or poorly defined usage. Motivated by the opportunity to gather a large number of contributing authors with the launch of this special issue on advanced MRS methodology, this article aims to describe many of these terms, to pinpoint differences in their meanings and to suggest specific uses or definitions.

This discussion applies to in vivo MR measurements in humans and animals obtained with single‐voxel spectroscopy (SVS, acquisition of a spectrum from a single localized volume) and MR spectroscopic imaging (MRSI, simultaneous acquisition of separate spectra from multiple volumes, based on spatial separation of these volumes by use of signal evolution under MR gradients). Not all terms in use could be included; some were thought to be obvious from common usage,^1^ particularly in MR imaging (MRI)^2^, ^3^ or high‐resolution NMR,^4^, ^5^ and were thus not added by intention, while others may still be evolving. To describe in detail what individual terms mean and how they differ from similar notation used in the literature, this work necessitated including descriptions of the basic concepts of MRS background, acquisition and processing methodology, as well as evaluation and quality assessments. To go deeper into understanding those concepts, the reader is referred to the original literature or to textbooks on MRS.^6^

This article is structured in five parts dealing with terminology for characterization of MR spectra (section 2), for description of acquisition methods (section 3), for use in data processing and quantification (section 4), for reporting of MR hardware (section 5), and for various further topics (section 6). Within these parts the terms are ordered, where possible, in a logical bottom‐up arrangement, not in alphabetical order. All terms are highlighted in bold where they are explained, not where they first appear, and potential abbreviations are introduced.

The main recommendations on terminology and concepts in in vivo MRS emerging from this paper are summarized in Table 1 and all terms mentioned in the text are collected in Table 2, supplemented by Table 3, which collects abbreviations for MRS‐specific pulse sequences and software that are sometimes used in the MRS literature without proper referencing.

**TABLE 1:** Summary of major recommendations for MRS terminology and concepts

(A) Terminology
1. The TD **signal amplitude** is also referred to as **signal intensity** and corresponds to the **signal (or peak) area** in FD. The FD terms **peak intensity** and **peak amplitude** are equivocal, either referring to peak area or peak height. If used, their meaning should be specified, or **peak height** should be used instead.
2. **Noise** should refer to random fluctuations in the acquired data only. Artifactual signal (eg, spurious echoes, leaking RF signal from other sources, outer volume signal bleed, deviation from ideal signal behavior due to instabilities) should not be referred to as noise.
3. **SNR** should be defined as signal per **one** SD of the noise. If the older convention of signal per two SD is used then it has to be declared explicitly.
4. The term **spectral resolution** should not be used to represent the digital resolution, ie, the frequency spacing between points in the spectrum. Spectral resolution represents the ability to separate partially overlapping spectral features (see below).
5. The term **baseline** should be used exclusively to describe nuisance signal underlying the metabolite spectra of interest. It should not be used to refer to signal components from mobile macromolecules, which are best referred to as **signals from macromolecules** or **macromolecular background** signals.
6. **Water (signal) suppression** (**WS**) **efficiency** should be quantified by the ratio of the residual water peak height in the water‐suppressed spectrum relative to the amplitude of the unsuppressed water signal in a reference acquisition acquired with otherwise identical acquisition parameters.
7. The **residual water signal** should be characterized by the height of the remaining water signal relative to the tallest metabolite peak in the water‐suppressed spectrum.
8. **Quality of volume selection** is characterized by
8.1. **selection efficiency**: actually selected signal from within VOI vs. totally available signal from within VOI;
8.2. **suppression efficiency**: 100% minus actually selected signal from outside VOI vs. totally available signal from outside VOI;
8.3. **contamination**: total selected signal from outside VOI vs. total selected signal from inside and outside VOI.
9. **Characterization of editing** experiments:
9.1. **Editing efficiency:** ratio of the integral of absolute values under the edited resonances (with relaxation‐weighting removed) to the same measure of those resonances in a spectrum acquired with zero echo time.
9.1.1. The **theoretical editing efficiency** refers to a theoretical value calculated or simulated for idealized experimental conditions.
9.1.2. The **practical editing efficiency** refers to the actual value obtained for a specific practical implementation and experimental setting.
9.2. **Editing yield:** signal retention after editing (including signal loss due to relaxation and other effects) relative to signal obtained without editing for the whole metabolite spectrum under best achievable conditions for the same in vivo situation.
10. The differentiation between **TD and FD fitting methods** is based on the domain in which the cost function is calculated and not on whether the signal model is formulated in TD or FD.
11. **Repeatability vs. reproducibility**: the term repeatability is used if measurements are repeated in exactly the same setting with the same operator and under identical conditions, while reproducibility refers to repeated measurements under different settings, eg, another method (methods comparison), operator (inter‐observer variance), another instrument (scanner or manufacturer dependence), site, or field strength.
12. The terms **chemical shift imaging (CSI)** and **MR spectroscopic imaging (MRSI)** have been used synonymously. We recommend using MRSI rather than the somewhat historic term CSI. The term spectral imaging should not be used.
13. At present, clinical MR scanners are categorized as **low field** for 1.5 T and below, **high field** for 3 T, and **ultrahigh field** for 7 T and above.

(B) Concepts
1. Determining **noise** in the residual signal from model fitting eases the problem of baseline signal from neighboring peaks but is not recommended for spectral areas with detectable metabolite resonances because residues from imperfectly fitted resonances might thus be included in the noise calculation. Using a signal‐free area of the measured spectrum (or the residual signal) for quantification of noise is encouraged.
2. Indications of **SNR** have to be accompanied by explanations on whether they refer to TD or FD SNR, what method was used to determine it (including the length of the acquired FID), which peak it refers to and, if based on FD peak height, whether the height includes underlying resonances or baseline.
3. The methyl peak of the creatines is best for **calibration of the chemical shift scale** for ^1^H MRS of brain (if present in sufficient intensity in the spectrum) and **indication of metabolite linewidth**.
4. **Spectral resolution**
4.1. Spectral resolution and linewidth should be defined in the form of **full width at half maximum** (FWHM).
4.2. Determination of FWHM is most robust for the water peak. If a water spectrum is available it is recommended to use water FWHM alone or in combination with a specifically defined metabolite peak width.
4.3. If no non‐suppressed water signal is available, FWHM from the fit for specific metabolites should be indicated.
5. **Spatial resolution** in MRSI: it is recommended that the definition of spatial resolution used is described in publications: ie, for the nominal voxel volume and/or effective voxel volume, either without or with interpolation. Specification of the effective voxel volume is strongly encouraged.
6. The **uncertainty of the estimated parameters (fit error)** must be estimated and listed for all interpreted parameters in publications. CRLB represent valid estimates for the lower bounds of the fit error. However, it should be noted that fit uncertainties are calculated differently in different fit packages and may thus differ systematically.
7. **CRLB** are sometimes used as indicators for a lower estimate of repeatability or even reproducibility, but it should be clear that they are not a valid replacement for estimates from repeatability or reproducibility studies.
8. It is recommended that fit packages include in their output the **fit quality number** (FQN) or a similar measure representing the amount of signal that is not represented by the fit, ie, the quality of the model fit.

**TABLE 2:** Terms mentioned and put into perspective in this contribution, including abbreviations used in this paper

Term listed in this paper	Abbreviation used in this paper	Reference to explanations in the text (sections)	Comment
absolute quantification		4.2.3	
absolute units		4.2.3	
absorption spectrum		2.1	
accuracy		4.3	
acquisition		3; 6	
agreement		4.3	
apodization		4.1.1	
apparent T_2_		2.3	
a priori information		4.2.1	
B_0_ shim systems		5.1	
B_1_^+^ amplitude		3.2	intensity of the time‐dependent transmit magnetic field
background signal		2.4	in the context of MM signals
background signal		6	in the context of isotope studies
bandwidth	BW	3.2	
baseline		2,4	
baseline correction		4.1.1	
carrier frequency		6	
chemical exchange		3.5	
chemical exchange saturation transfer	CEST	3.5	
chemical shift		2.2	
chemical shift displacement error	CSDE	3.2	
chemical shift imaging	CSI	6	equivalent meaning as MRSI, not a recommended term
chemical shift encoded MRI		6	MRI technique using multiple gradient echoes. Techniques that should not be termed CSI or MRSI to prevent confusion
cerebrospinal fluid	CSF		
choline	Cho		
co‐editing		3.4	
coherent image artifacts		3.3	
coil combination		4.1.1	
compartment effects		4.2	
compressed sensing		4.1	
confidence limits		4.2.2	
contamination		3.2	
continuous wave	CW		
cost function		4.2.1	
COSY		3.4	correlation spectroscopy
Cramér‐Rao lower bounds	CRLB	4.2.2	
Cramér‐Rao minimum variance bounds	CRMVB	4.2.2	equal in meaning, but less frequently used than CRLB
creatine	Cr		
decoupling		6	
digital filtering		4.1.1	
dipolar coupling		2.2	
dispersion spectrum		2.1	
downfield		2.2	
dwell time		3.1	
echo		2.1	
ectopic lipids		2.5	
eddy‐current correction	ECC	4.1.1	
editing		3.4	
editing efficiency		3.4	the theoretical and the experimental editing efficiencies should be clearly distinguished
editing yield		3.4	
effective voxel volume		3.3	
elimination of spurious signals		4.1.1	
error estimation		4.2.2	
exam repeatability		4.3	
excitation		6	
extended k‐space sampling		3.3	
external standards		4.2	
extracranial lipids		2.5	
FD fitting method		4.2.1	
field of view	FOV	3.3	
first‐order phase correction		4.1.1	
fit error		4.2.2	
fit quality number	FQN	4.2.1	
flip angle	FA	3.2	
free induction decay	FID	2.1	
frequency adjustment		5.2	
frequency correction		4.1.1	
frequency domain	FD	2.1	
frequency instability correction		4.1.1	
full width at half maximum	FWHM	2.3	
functional MRS	fMRS	6	
gamma‐aminobutyric acid	GABA		frequently erroneously expanded as gamma‐aminobutyrate. However, near neutral pH, the molecule predominantly exists as the zwitterionic form of the acid, while butyrate would imply the deprotonated form of gamma‐aminobutyric acid
Gaussian lineshape		2.1	
glutamate plus glutamine	Glx		Glu + Gln
glycerophosphocholine	GPC		
gradient performance		5.1	
gray matter	GM		
grid shifting		4.1	
half‐echo sampling		2.1	
heteronuclear		6	
high‐field scanner		5.1	
homonuclear		6	
hyperparameters		4.1.1	
hyperpolarized		3.5	
image blurring		3.3	
incoherent image artifacts		3.3	
institutional units		4.2.3	
interpolated voxel volume		3.3	
inter‐subject differences		4.3	
intrinsic T_2_		2.3	
inversion transfer		3.5	
isotope delivery studies		6	isotope infusion studies in case of intravenous isotope administration
*J*‐coupling		2.2	
*J*‐difference editing		3.4	
*J*‐evolution		2.2	
*J*‐modulation		2.2	
*J*‐resolved		3.4	
k‐space apodization		4.1	
k‐space pattern		3.3	
k‐space undersampling		3.3	
k‐space weighting		3.3	
least squares model fitting		4.2.1	
linear combination model fitting		4.2.1	
lineshape transformation		4.1.1	
lineshape type		2.1	
linewidth		2.3	
lipid signal reduction		4.1	
lipid signals		2.5	
localization gradient		3.2	
longitudinal relaxation		3.5	
longitudinal relaxation enhancement		3.5	
Lorentzian lineshape		2.1	
low‐field scanner		5.1	
macromolecules	MM	2.4	MM can also mean macromolecular, or mobile macromolecules
macromolecular background signal		2.4	
magnetic field strength		5.1	
magnetic resonance	MR	1	
magnetization transfer	MT	3.5	
maximum likelihood parameter estimation		4.2.1	
MEscher–GArwood	MEGA		
metabolite ratio		4.2.3	
mobile lipids		2.4	
molal	m	4.2.3	moles per mass of solvent
molar	M	4.2.3	moles per volume of tissue
moles per mass of tissue		4.2.3	moles per kilogram, often per kilogram wet weight
motion correction	MOCO	6	
MR imaging	MRI	1	
MR spectroscopic imaging	MRSI	3.3; 6	
MR spectroscopy	MRS	1	
MR visibility		4.2	
MRS pulse sequence		2.1	
MRSI reconstruction		4.1	
multiple‐quantum‐coherence filtering	MQF	3.4	
N‐acetylaspartate	NAA		
N‐acetylaspartylglutamate	NAAG		
natural abundance		6	
noise		2.1	
nominal voxel volume		3.3	
nuclear magnetic resonance	NMR	1	
number of (sampling) points	NP	3.1	
number of averages	NAV	6	
number of excitations	NEX	6	
nuclear Overhauser enhancement	NOE		
on resonance		6	
orientation dependence		6	
outer volume signal bleed		2.4	
outer volume suppression	OVS	3.2	
parameter estimation		4.2.1	
parallel imaging		4.1	
partial‐echo sampling		2.1	
partial volume contributions		4.2	
parts per million	ppm		
pass‐band		3.2	
peak B_1_^+^		5.1	often simplified to peak B_1_
peak height		2.1	
peak integration		4.2.1	
phase cycling		3.2	
phase encoding		3.3	
phase instability correction		4.1.1	
phosphocholine	PCho		often also abbreviated as PC or PCh
phosphocreatine	PCr		
physiologic motion		6	
point spread function	PSF	3.3	
polarization transfer	PT	3.5	
postprocessing			
precision		4.3	
preprocessing		4.1	
prescan		5.2	
prior knowledge		4.2.1	
prospective drift correction		4.1.1	
prospective motion correction	MOCO	6	
pulse calibration		3.2	
pulse duration		3.2	
pulse shape		3.2	
quality of model fitting		4.2.1	
quantification		4.2; 4.2.3	
quantitation		4.2; 4.2.3	
radiofrequency	RF		
ratio to water signal		4.2.3	
real‐time adjustments		4.1.1	
receive coils		5.1	
receiver gain		5.2	
recovery rate		6	
recovery time constant		6	
region of interest	ROI	3.2	
regridding		4.1	
relative tissue volume fractions		4.2	
relaxation corrections		4.2	
relaxation rates		4.2.1	
reliability		4.3	
repeatability		4.3	
repetition		6	
reproducibility		4.3	
residual dipolar coupling		6	
residual lipid signal		2.5	
residual signal		2.1	
residual water signal		2.5	
resolution enhancement		4.1.1	
resonance line		2.1	
RF pulse type		3.2	
sampling interval		3.1	
sampling trajectory		3.3	
saturation transfer		3.5	
scalar coupling		2.2	
scalar coupling evolution		2.2	
scan‐to‐scan repeatability		4.3	
selection efficiency		3.2	
shimming		5.2	
short‐term repeatability		4.3	
shot		6	
signal amplitude		2.1	
signal bleed		3.3	
signal contamination		3.2	
signal intensity		2.1	
signal‐to‐noise ratio	SNR	2.1	
single average		6	illogic expression
single recording		6	
single scan		6	
single‐voxel spectroscopy	SVS	1	
singular value decomposition	SVD	4.1.1	
slice profile		3.2	
slice profile imperfections		3.2	
slice thickness		3.2	
sources of variance		4.3	
spatial filter		4.1	
spatial interpolation		4.1	
spatial registration		4.1	
spatial response function	SRF	3.3	
spatial‐spectral encoding		3.3	
spectral editing		3.4	
spectral resolution		2.3	
spectral width	SW	3.1	
spoiler gradient pulses		3.2	
spurious echoes		2.5	
static magnetic field	B_0_	5.1	
stop‐band		4.2	
suppression efficiency		2.5	
susceptibility shift		6	
TD fitting method		4.2.1	
tetramethylsilane	TMS		
time domain	TD	2.1	
tissue concentration		4.2.3	often just referred to as concentrations, but intended to stand for metabolite content expressed as average value over multiple tissue compartments, to be distinguished from chemical concentrations in homogeneous solutions
total NAA	tNAA		NAA + NAAG
total creatine	tCr		Cr + PCr
total choline	tCho		sum of all choline species
trace		6	
transient		6	
transition‐band		4.2	
transmit coils		5.1	
transmit power adjustment		5.2	
transverse relaxation		2.3	
two‐dimensional spectroscopy		3.4	
type of spatial selection		3.2	
ultrahigh‐field scanner		5.1	
uncertainty of the estimated parameters		4.2.2	
units to express results		4.2.3	
upfield		2.2	
Voigt lineshape		2.1	
volume of interest	VOI	2.1	
water presaturation power adjustment		5.2	water signal presaturation power adjustment
water saturation parameters		5.2	water signal saturation parameters
water suppression	WS	2.5	water signal suppression
water suppression efficiency		2.5	water signal suppression efficiency
white matter	WM		
zero‐filling		4.1.1	
zero‐order phase correction		4.1.1	
zero‐padding		4.1.1	alternative, but not recommended term for zero‐filling

**TABLE 3:** List of abbreviations for MRS‐specific pulse sequences and software that are sometimes used in the MRS literature in a stand‐alone fashion without explanation or reference. For more detailed explanations, refer to the original references listed or the monograph by de Graaf^6^, which contains an extended list of abbreviations, or to other textbooks

Abbreviation	Full name	Explanation	Ref(s)
AMARES	Advanced Method for Accurate, Robust and Efficient Spectral fitting	Time domain fitting method for MRS data with use of prior knowledge constraints, implemented in jMRUI	^7^
CHESS	CHEmical Shift Selective (water suppression)	Water suppression technique using frequency‐selective pre‐pulses	^8^, ^9^
EPSI	Echo‐Planar Spectroscopic Imaging	fast MRSI sequence making use of data sampling under oscillating readout gradients between traditional spectroscopic data samples to simultaneously encode the spectral and one spatial dimension	^10^, ^11^, ^12^
ISIS	Image‐Selected in vivo Spectroscopy	Multi‐shot SV localization sequence based on 8 acquisitions with different combinations of slice‐selective inversion pre‐pulses	^13^
jMRUI	Java‐based Magnetic Resonance User Interface	Software package for processing and fitting MR spectroscopy data using TD models and cost functions defined in TD	^14^, ^15^
LASER	Localization by Adiabatic Selective Refocusing	Single shot SV localization sequence based on a spin echo from 6 slice‐selective adiabatic refocusing pulses after initial adiabatic excitation of the whole sample	^16^, ^17^, ^18^
LCModel	Linear Combination Model (fitting package)	Commercial model fitting package based on modeling the in vivo spectrum as a combination of known basis spectra	^19^
PRESS	Point‐RESolved Spectroscopy	Single shot SV localization sequence based on a spin echo from 2 slice‐selective refocusing pulses after initial excitation of a slice using a slice‐selective 90° pulse	^20^, ^21^
Semi‐LASER	Semi‐Localization by Adiabatic Selective Refocusing	Single shot SV localization sequence based on a spin echo from 4 slice‐selective adiabatic refocusing pulses after initial excitation of a slice using an amplitude‐modulated slice‐selective 90° pulse	^22^
STEAM	Stimulated Echo Acquisition Mode	Single shot SV localization sequence based on a stimulated echo from 3 slice‐selective 90° pulses	^23^, ^24^, ^25^
SPECIAL	SPin‐ECho‐full‐Intensity‐Acquired‐Localization	SV localization sequence using 2 acquisitions based on a spin echo with slice‐selective pulses and an ISIS element with 1 slice‐selective inversion pre‐pulse for the third direction	^26^
VAPOR	VAriable pulse Power and Optimized Relaxation delays	Water suppression technique using frequency‐selective pulses of optimized flip angles and delays to be insensitive to changes in relaxation times and power calibration inaccuracies	^27^
WET	Water suppression Enhanced through T_1_ effects	Water suppression technique using multiple frequency‐selective prepulses with optimized flip angles to be less sensitive to B_1_ and T_1_ changes	^28^

## 2 TERMINOLOGY FOR THE CHARACTERIZATION OF MR SPECTRA

### 2.1 Signal and noise

The **MRS signal** *S*(*t*) is acquired in the **time domain** (TD) and refers to the induced voltage in receive coils as response to precession of transverse magnetization generated by an ensemble of nuclear spins present in a biological sample or in situ tissue. The acquired signal is proportional to the transverse magnetization generated by an MRS pulse sequence consisting of one or multiple radiofrequency (RF) pulses and field gradient pulses that interact with the spin systems within the sample. In the absence of a read‐out field gradient (or fields related to eddy currents; see below) as well as T_1_‐saturation effects, and in response to a simple excitation (equivalent to a single excitation pulse), *S*(*t*) can be described broadly as a sum of exponentially and Gaussian‐damped, complex‐valued sinusoidal model functions *M*_*j*_(*t*) and a noise term *ε*(*t*) ^7^, ^8^, ^9^:

(1) $$S\left(t\right)=M\left(t\right)+\varepsilon \left(t\right)=\sum_{j=1}^{L}A_{j}\exp\left(-i\omega_{j}t+i\phi_{j}-\frac{t}{\;T_{2,j}^{*}}-\frac{t^{2}}{T_{G,j}^{2}}\right)+\varepsilon \left(t\right).$$

In this form, *S*(*t*)is also called a **free induction decay** (FID), in contrast to signal that increases before it decays, which is termed an **echo** and where the signal can be recorded with **half‐echo sampling** (FID‐like, start of data sampling at top of the echo or **partial‐echo sampling** when the start occurs earlier, usually as soon as possible after preceding RF and gradient pulses). Each **resonance line** *j* of an MR spectrum is thus characterized by five model parameters, its amplitude *A*_*j*_, its resonance frequency *ω*_*j*_, its phase *ϕ*_*j*_, its transverse relaxation time $T_{2,j}^{*}$ and its Gaussian‐damping constant *T*_*G,j*_. With $\frac{1}{\;T_{2,j}^{*}}\neq 0$ and $\frac{1}{T_{G,j}^{2}}=0,$ the **lineshape type** is **Lorentzian**; with $\frac{1}{\;T_{2,j}^{*}}=0$ and $\frac{1}{T_{G,j}^{2}}\neq 0$ **Gaussian**; and if both terms are different from zero it is of type **Voigt**. (For more complicated lineshapes, see section 2.3, Linewidth.) The TD **signal amplitude** *A*_*j*_ is also referred to as **signal intensity** and corresponds to the **signal (or peak) area** in the **frequency domain** (FD) description of the MR signal obtained by Fourier transformation (FT). The terms **peak intensity** and **peak amplitude** are ambiguous (usually referring to peak area) and should not be used to mean **peak height** in FD. With *ϕ*_*j*_ *=* 0 and upon FT the real part of the data is called the **absorption spectrum** and the imaginary part the **dispersion spectrum**.

In MRS, **noise** should refer to random fluctuations in the acquired data caused by thermal variations in the investigated object or the detection hardware. Artifact contributions to the signal (eg, spurious echoes, leaking RF signal from other sources, outer volume signal bleed, deviation from ideal signal behavior due to instabilities) should not be referred to as noise. In the real and imaginary part of MR spectra noise has uniform spectral power (white noise) and follows a Gaussian intensity distribution, while it has Rician characteristics in magnitude display. These characteristics should be respected when generating artificial noise for synthetic MRS signals. Manufacturers’ and users’ processing steps (eg, analog and digital filters, apodization, or regularized machine‐learning and prior‐knowledge‐based reconstruction) may alter the noise distribution. The noise amplitude σ, defined as the standard deviation (SD) of the data in signal‐free areas, can be determined in either TD or FD (ie, in TD, σ^TD^ corresponds to the SD of *ε(t)* from Equation 1). For the standard definition of the FT (non‐symmetric scaling in forward and reverse FT) and regular sampling, the two measures are related for a signal with n points by:

(2) $$\sigma^{\mathrm{FD}}=\sigma^{\mathrm{TD}}\sqrt{n}$$

For measurements of noise in vivo, it is recommended to use a spectral range without inherent signals rather than **residual signal** in the fitted spectral range after model fitting (ie, experimental signal minus modeled signal). For ^1^H‐MRS, spectral ranges of the absorption mode spectrum <−1 or >9 ppm (or the signal‐free tail of the TD signal) are recommended after any possible tail of neighboring resonances has been approximated with a linear signal estimation and subtracted. If there are indeed no other signal contributions other than from noise (in particular also no offset from zero mean amplitude, often caused by a bias in the first point in the FID), the root mean square (rms) value is identical to the SD, or else the rms value that is used in some software packages is an overestimate of the true noise in terms of σ. The noise amplitude is equal in both channels of the complex data, thus determination in one channel may be sufficient.

The **signal‐to‐noise ratio** (SNR) is the most widespread criterion for assessment of MRS data quality. It is therefore paramount to use an unequivocal definition for this measure. Although in most disciplines SNR is defined as signal per one SD of the noise, historically a definition as signal per two SD has been widespread^10^ and is still in practice † in MRS/NMR. Today, the standard physics definition has also become more common in NMR and MRS literature. Hence, we strongly suggest uniformly using the definition of

(3) $$\mathrm{SNR}=\text{Signal}/\sigma_{\text{noise}}$$

If another definition is used for historical reasons then it must be declared.

Both signal and noise can be measured in either TD or FD, which result in very different values and represent different properties of the data. For comparisons, they should be expressed in the same domain.

There are many options for choosing the most appropriate parameter of the acquired data as signal. In TD, it is customary to use the magnitude of the first point in the FID, which correlates with the area under the whole spectrum in FD. However, it also includes residual unsuppressed water or lipid signal in ^1^H MRS and thus is not useful as a measure of spectral quality straight away. The initial point of the FID can be affected by analog and digital filtering in the receive chain (including that it may be halved or averaged with the terminal point of the FID to eliminate baseline problems^34^). In low‐SNR cases and if there are substantial filtering effects, it may be reasonable to model the initial part of the FID to back‐extrapolate the first point for SNR calculation. In order to define a metabolite‐specific SNR, one can use the fitted metabolite area (either from that fit in TD or FD), which corresponds to the first point in time for the metabolite‐specific response. With all these definitions in TD, SNR is independent of linewidth (and shimming).

In FD, signal can refer to peak height of the unsuppressed water signal, largest metabolite peak of interest or a specific (frequently singlet) peak of interest in the spectrum. This makes FD‐SNR depend on linewidth and thus provide a mixed measure reflecting relative signal strength and spectral resolution achieved. The most robust measure uses either the unsuppressed water signal^35^ or largest metabolite peak height in the measured magnitude spectrum, which eliminates variance due to incorrect phasing, baseline definition or influence of fit model. Conversely, the most meaningful measure of signal would refer to the peak height of a fitted metabolite peak in pure absorption mode excluding the baseline and overlapping signals. However, this definition relies on model fitting, the appropriateness of the model and the availability of peak height from the fit package. Very widespread is the use of signal as measured peak height in the phased absorption mode spectrum including the baseline and underlying background signals. Most often in brain ^1^H MRS, the largest metabolite peak is reported, often excluding lipid signals as found in tumors, while in body MRS the lipid resonances may be the targeted spectral components. Alternatively, since peak area is usually available numerically from fitting rather than peak height, and also to obtain a measure of signal intensity that is independent of linewidth, it has been suggested to use fitted metabolite peak area per SD of noise, which essentially corresponds to the metabolite‐specific TD SNR defined above.

**FIGURE 1:**
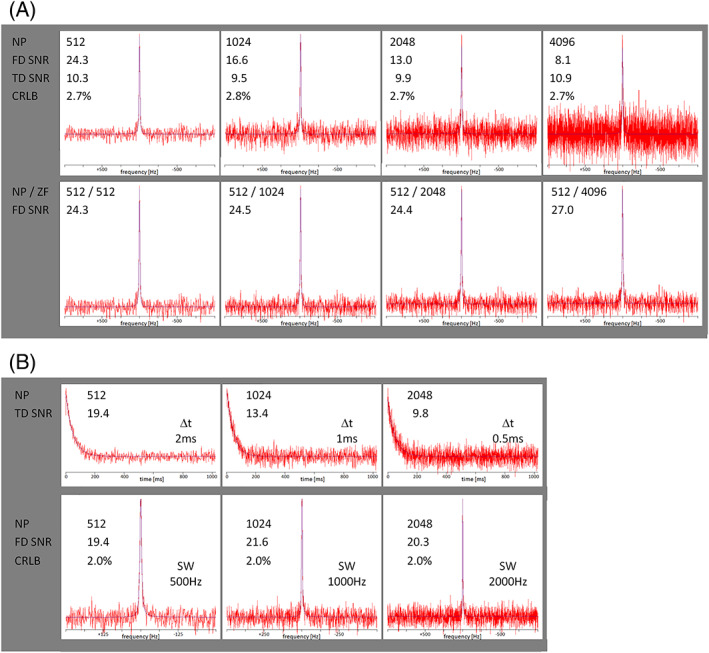
Illustration that demonstrates how the length of acquisition and sampling rate influence SNR in TD and FD, as well as CRLB. (A) shows in the upper trace the decrease of FD SNR (based on peak height) with length of actual acquisition (more acquired data points with identical sampling rate) while the TD SNR and the fit uncertainty (CRLB) essentially remain the same. By contrast, the lower trace documents that extension of the FID by adding zeroes (zero‐filling) does not alter the FD SNR (TD SNR is then ill‐defined and calculation of CRLB would have to include a zero‐filled signal model, leading to unchanged CRLB). (Simulated single Lorentz line with constant amplitude and width [12.6 Hz], changing noise). (B) illustrates the effect of changing the sampling rate (at constant duration of signal acquisition), which brings along a change in analog and/or digital filters causing high‐frequency noise to be excluded from the acquired FID and thus higher TD SNR at smaller bandwidths. FD SNR and CRLB for the amplitude of the single line remain unchanged. (Simulated single Lorentz line with constant amplitude and width [6.3 Hz], created from identical data set by digital filtering). Δt, sampling interval; NP, number of sampled points; SW, spectral width; ZF, number of points after zero‐filling

When reporting SNR, it is crucial to specify the measurement method and for which peak it is determined. In addition, FD noise, and hence FD‐SNR, depends crucially on the length of the TD signal (where FIDs extending beyond the decayed metabolite signals only increase spectral noise). To allow for meaningful comparison of SNR values, it is thus absolutely required to report the length of the FID or sampling rate (inverse of spectral bandwidth) plus the number of acquired time points along with the SNR value. This is illustrated in Figure 1. To accurately define peak height, the peaks have to be sampled with a sufficient number of points in FD; otherwise the height can vary substantially with small frequency shifts. Thus zero‐filling (see more detailed information in section 4.1.1) is appropriate to determine FD‐SNR if the signal has decayed sufficiently at the end of the FID such that it does not create ringing artifacts; in this case, it does not alter the SD of the FD noise.

When comparing the (SNR) performance of acquisition sequences, care should be used to make sure the size and shape of the localized **volume of interest** (VOI) is identical (or corrected for by using SNR per actually selected volume, including the actual shape of the VOI), and it may be of interest to compare SNR per unit time (ie, SNR scaled by the square root of the total acquisition time taken by a single experiment) rather than per acquisition. For MRSI, the final effective voxel volume (as defined in section 3.3) should be taken into account.

### 2.2 Chemical shift and couplings

The **chemical shift** refers to the frequency scale relative to a frequency reference. It is expressed in parts per million (ppm) to be independent of magnetic field strength (B_0_). In high‐resolution ^1^HNMR, the signal of tetramethylsilane (TMS) was proposed as the standard to define 0 ppm. ‡ In in vivo MRS, the chemical shift scale is calibrated with any well‐identified known resonance peak in the spectrum. Since all major singlet peaks in ^1^H MRS of brain are composite peaks (N‐acetylaspartate [NAA] and N‐acetylaspartylglutamate [NAAG]; creatine [Cr] and phosphocreatine [PCr]; choline [Cho], phosphocholine [PCho] and glycerophosphocholine [GPC]), none of them is ideal as frequency reference. However, given that the difference between the subcomponents is smallest for the methyl peak of the creatines, this is the recommended peak for calibration of the chemical shift scale when available with enough SNR. In accordance with the widely used spin system parameters tabulated elsewhere,^36^, ^37^ it should be set to 3.028 ppm. In cases of pathology, other peaks may be preferable (eg, the trimethylammonium peak of the choline‐containing compounds in ^1^H MRS of tumor). In ^31^P NMR, the conventional primary reference standard is 85% phosphoric acid to define 0 ppm. In vivo, the PCr or alpha ATP resonance is used to calibrate the chemical shift scale. In concordance with the NMR convention, the PCr peak in tissue at physiologic rest conditions would then be set to −2.35 ppm. However, it has become customary for ease and robustness of use to set the PCr resonance to 0 ppm in in vivo studies. Care should therefore be used when contrasting chemical shift values from different publications, in particular when comparing pH or ion‐concentration–sensitive peak positions (see, eg, reference^38^ for chemical shifts of many phosphorus‐containing compounds and their pH dependence).

**Upfield, downfield direction in MR spectra**: by convention based on continuous wave (CW) NMR, the increasing chemical shift axis (plotted towards the left) points in the downfield direction and lower chemical shift values refer to the upfield direction. In ^1^H MRS, the spectral range with chemical shifts higher than water is often termed the downfield region (ie, >4.7 ppm), whereas the range with lower chemical shift values is called the upfield range.

***J*‐coupling (synonymously termed scalar coupling)** stands for the interaction (expressed in Hz) between nearby nuclear magnetic moments mediated by electron spins. The strength of the *J*‐coupling interaction generally decreases as the number of bonds between the coupled nuclei increases, and it generally becomes negligible (*J* << linewidth) for more than three bonds. *J*‐coupling results in splitting of resonances into multiplets, as well as echo‐time‐dependent modulation of peak phase and amplitude (called ***J*‐modulation**, ***J*‐evolution**, or **scalar‐coupling evolution**).

**Dipolar coupling**: direct (through space) spin–spin interaction that is relevant for relaxation and relayed magnetization transfer (MT), but does not lead to peak splitting in the case of fast and isotropically tumbling molecules.

### 2.3 Linewidth

**Spectral resolution** is one of the most important factors affecting the reliability of metabolite quantification and represents the ability to separate partially overlapping spectral features. The term “spectral resolution” should not be used to represent the digital resolution, ie, the frequency spacing between points in the spectrum. A proxy for spectral resolution is the resonance **linewidth**, which is commonly defined as the **full width at half maximum** (FWHM) of a singlet resonance measured digitally in the FD in Hz (or ppm) at half maximum height. Its correlate in TD is the decay constant, but that cannot be easily determined experimentally for an FID with multiple resonances and must also account for lineshape. The minimum resonance linewidth under in vivo conditions is determined by the transverse relaxation (natural linewidth, 1/(πT_2_), due to exponential T_2_ damping §) and microscopic susceptibility variation originating from the cellular tissue structure.^39^, ^40^ ¶ Such a spectral resolution is observed experimentally only if the macroscopic B_0_ inhomogeneity over the VOI is sufficiently removed by B_0_ shimming.^41^ With poor B_0_ shimming or B_0_ inhomogeneities that cannot be approximated by the available shim terms, the resonance linewidth increases and the FD lineshape can be of arbitrary asymmetric form that cannot be accurately modeled by exponential and Gaussian decay terms in TD and usually leads to a bias in estimated parameters. The final spectral resolution can be further altered by the effects of eddy currents and shot‐to‐shot variations in frequency and phase if signal averaging is used and these fluctuations are not removed in preprocessing. In high‐resolution NMR and in MRI, the signal decay is often described by an exponential model, where the nonreversible T_2_ decay is extended by a T_2_^+^ decay to add up to a so‐called T_2_^*^ decay (1/T_2_^*^ = 1/T_2_ + 1/T_2_^+^). T_2_^+^ entails all decay contributions that can be removed by refocusing of the transverse magnetization. However, T_2_ is sometimes also called apparent transverse relaxation time (**apparent T**_**2**_) since it includes dynamic dephasing effects and its measured value thus depends on the dynamic rephasing properties of the pulse sequence used.^42^ By contrast, what is called **intrinsic T**_**2**_ excludes diffusion‐related dephasing effects and represents the dephasing effects of the homonuclear dipole–dipole interaction, the hyperfine contact interaction with paramagnetic centers, and cross‐relaxation.^42^

It is recommended that the spectral resolution be described in the form of FWHM. Since baseline effects and signal overlap complicate the interpretation of metabolite FWHM values (and if taken from fitting programs, they depend on the fitting model), FWHM obtained from unsuppressed water is the most robust indicator of spectral resolution for ^1^H MRS. It should be noted that this will generally be different from the metabolite linewidth due to the shorter T_2_ of water compared with the singlets of NAA or Cr. By contrast, a substantial inclusion of cerebrospinal fluid (CSF) with long inherent T_2_ may mimic better resolution. For MRS outside the nervous system the linewidth of water may not be a very sensitive or trustworthy indicator of shim quality, because water linewidths are intrinsically wide (short T_2_ in muscle and liver) and can be influenced by variable iron content of the organ (liver). If FWHM values are calculated from magnitude, rather than absorption spectra, this must be specified since they are considerably larger (by √3 for Lorentz, Gauss and Voigt lines). Furthermore, obtaining non‐water‐suppressed MRSI data is time‐consuming and thus the FWHM taken from spectral fitting of the metabolite signals may frequently be the best option for MRSI. Note that the difference in the FWHM of the water and metabolite resonances due to their T_2_ values will depend on B_0_, pathology, and the organ under investigation. Furthermore, a certain FWHM may represent different degrees of spectral overlap depending on the lineshape, when deviating from Lorentzian (see Frequency and phase instability correction in section 4.1.1, for how to prevent line broadening from signal averaging).

### 2.4 Baseline and background

The **baseline** of in vivo ^1^H MR spectra consists of smoothly varying signal components underlying the inherent resonance peaks from the VOI, ie, those spectral features that would originate from the VOI under ideal acquisition conditions. Different phenomena may contribute to the baseline profile. Baseline contributions mostly originate from sub‐optimal localization performance. Non‐reproducible baseline features can be caused by unwanted resonances (water, lipids) from outside the VOI (**outer volume signal bleed**). Insufficient water suppression is another baseline source. Although water resonates outside the usually analyzed spectral range, the broad tail of the residual water signal, which is often out of phase, can substantially add to the baseline in the spectral region of interest. Apparent spectral baseline features can also be caused by hardware imperfections, specifically by corruption of the first few data points in the FID,^42^ and by residual eddy currents. Finally, a distorted or rolling baseline can originate from inaccurate timing of the echo relative to the start of data acquisition, necessitating strong first‐order phase correction. Artifact signals, like spurious echoes, may also underlie the inherent signals, but are usually not considered as part of the baseline, because they are usually not smoothly varying.

Reproducible broad signals originating from fast‐relaxing macromolecules (proteins) and mobile lipids (if present in brain pathology or most nonnervous tissues) should not be considered as baseline, but rather as **background signal from (mobile) macromolecules** (MM) and **lipids**, where the optional descriptor “background” is often used when the narrow lines from small metabolite molecules are the main targets of study, while the broad MM or lipid signals are presumed to provide a constant background signal. These signals are well characterized and are best treated as additional signal components for spectral fitting. (For details on MM signals, including the terminology of its components, see reference^44^).

### 2.5 Residual water and fat signal

**Residual water** or **residual fat signal** refers to remaining water/fat signals in spite of sequence modules aiming at their suppression. The **water (signal) suppression** (WS) **efficiency** can be specified by the ratio of the residual water peak height relative to the height of the unsuppressed water signal. The **residual water signal** is characterized by the height of the remaining water signal in the water‐suppressed spectrum relative to the tallest metabolite peak in the spectrum. Both measures are most robust when the water peak height is taken from magnitude spectra, although that can overestimate the signal for very good suppression efficiency. In addition, it can be helpful to indicate whether the residual water resonance is either in‐ or out‐of‐phase with the rest of the spectrum. Residual water signals from distant areas outside of the VOI can generate unwanted **spurious echoes** that will appear in the spectra as oscillating signals (wiggles), whereas if located along the read/phase encoding directions in MRSI they may also appear as broad smooth baseline signals at chemical shifts other than 4.7 ppm.^45^

**Lipid signals** observed in single‐voxel ^1^H MR brain spectra of healthy subjects usually result from subcutaneous lipid contamination because of imperfect VOI localization (therefore also termed **extracranial lipids**). They are typically out‐of‐phase and the largest contributions appear at ~1.5 ppm (depending on the B_0_ offset at the signal origin), and they also extend further downfield with multiple resonances between 2 and 5.5 ppm. Lipid signal contamination in MRSI is caused by the spatial response function and temporal instabilities (see below). Phospholipids of cell membranes are not detectable by in vivo ^1^H MRS. By contrast, **mobile lipids** are ubiquitous in health and disease in most organs except the nervous system. In nonfat tissue they are called **ectopic lipids**. In brain, they may be detectable in brain tumors, necrotic tissue, lipomas, or in patients with metabolic disorders, such as adrenoleukodystrophy,^46^ but usually only in specific populations and locations in healthy brain tissue.^47^

## 3 TERMINOLOGY FOR THE DESCRIPTION OF MR ACQUISITION METHODS

The first stages of MRS exams involve localization and creation of observable magnetization, followed by reception, amplification, frequency reduction by reference mixing, quadrature detection, bandpass filtering, and finally analog‐to‐digital conversion. Collectively, we attribute these signal manipulation steps to “acquisition”.

### 3.1 General terms

In data acquisition, **spectral width** (SW, in Hz) refers to the inverse of the **sampling interval** (or **dwell time**). There are often two spectral widths involved: one concerning the actual sampling rate of the analog‐to‐digital converter and another after digital **downsampling** (specifics often out of control for the operator) to the prescribed spectral width, which is reported in publications. The **number** of (sampling) **points** (NP #) refers to the number of complex points after downsampling, which allows calculation of the length of data acquisition (NP/SW) and thus the length of the original time‐domain signal (FID or echo). In the case of echoes, it should be made clear whether this refers to the full echo or a half echo.

### 3.2 Localization

The region of tissue that is selected for study by MRS is referred to as either the **region of interest** (ROI) or the **volume of interest** (VOI). These two terms have often been used interchangeably. Many spectroscopists prefer VOI to describe the localized spatial region in SVS and ROI for a selected area in MRSI. Because ROI is frequently used for specifically selected regions within an MRI or MRSI map in data evaluation, we recommend using VOI for the preselected regions in both MRS and MRSI acquisitions.

Spatial selection in SVS and prelocalization in MRSI is usually achieved by spatially selective RF pulses and is characterized by the following parameters:

The **type of spatial selection** can be based on excitation, refocusing, inversion (with add/subtract cycles), or outer volume suppression pulses and/or on coil receive sensitivity profiles.^48^, ^49^

RF pulse properties include the **RF pulse type**, which is usually called conventional if the pulse amplitude is described by a simple analytical formula (eg, Gauss‐weighted sinc) and the pulse is amplitude‐modulated only, ie, with constant carrier frequency. Among the frequency‐ (or phase‐) modulated RF pulses, adiabatic^50^ pulses are most widespread because they are less sensitive to any errors in the transmit field (B_1_^+^) caused by misadjustment or spatial inhomogeneity, due to an intra‐pulse frequency sweep and because they can achieve a much wider bandwidth for the same B_1_^+^ amplitude than amplitude‐modulated pulses. Depending on the simultaneously played field gradients, RF pulses can be selective in the spectral domain, the spatial‐spectral domain^51^ (ie, selective in spatial and spectral dimensions), or multiple spatial dimensions^33^ (2D or 3D).

**FIGURE 2:**
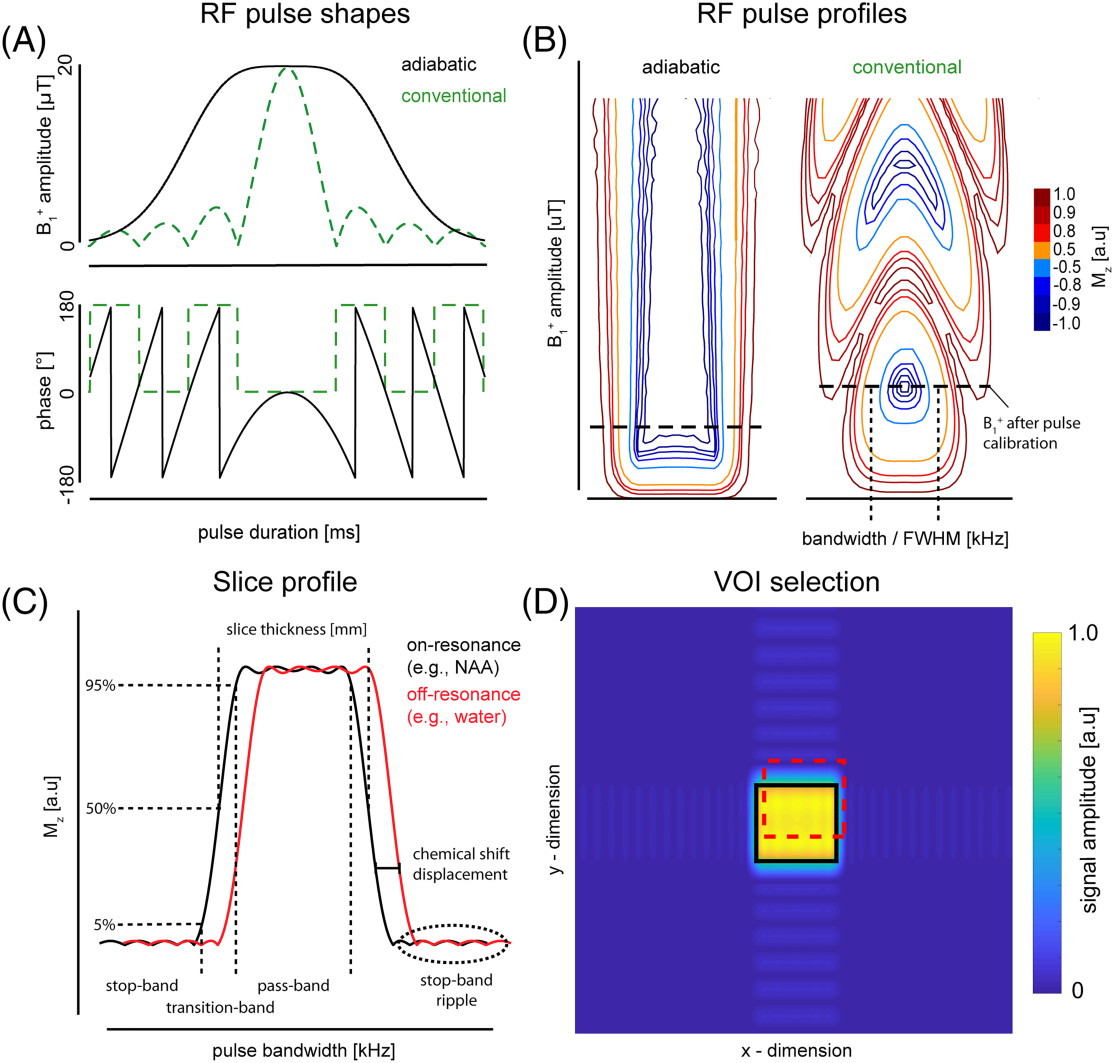
Illustration of terminology for RF pulses and spatial selection: RF pulses are characterized by (A) their RF pulse shape including a B_1_^+^(t) magnitude and phase envelope. For amplitude‐modulated RF pulses (constant carrier frequency, green dashed line), the phase envelope is constant except for 180° jumps (sign change of amplitude). For adiabatic pulses, the phase/carrier frequency envelope is time‐dependent (black solid line).Playing out an RF pulse translates into excitation/inversion of a certain frequency‐band (B). Ideally, only magnetization with a resonance frequency within this band is affected. In reality, the RF pulse profile (B) is nonideal (ie, not rectangular) as illustrated for an adiabatic (left) and a conventional inversion pulse (right). The bandwidth of RF pulses is defined by the FWHM of the pulse profile (frequency width at 50% of the profile maximum). The contour plots illustrate how strongly the magnetization M_z_ at different resonance frequencies is affected by the RF pulse as a function of the B_1_^+^ amplitude (−1: full inversion; +1: no effect).Playing out a B_0_ gradient concomitantly with an RF pulse translates the pulse profile into a slice profile (C). Both slice and pulse profiles can be further characterized by the pass‐band (frequency range over which the profile is >95% of its maximum), the stop‐band (<5% of the maximum) and the transition‐band (5% to 95% of the maximum). The shift of the slice profile for entities with inherently different resonance frequencies (ie, CSDE) is qualitatively illustrated in (C) (slice profile for an “on‐resonance” compound such as NAA in black vs. that for an “off‐resonance” compound such as water in red solid lines). (D) As the volume of interest (VOI) is often selected using different RF pulses for the different directions, the CSDE (spatial shift between the black solid and the red dashed rectangle), as well as the slice imperfections (indicated by the passband ripples and transition bands in (C)), can vary with direction (eg, for refocusing vs. excitation‐based selection)

RF pulses have a **pulse shape** (Figure 2A), which is defined by a time‐dependent amplitude, denoted B_1_^+^(t), and time‐dependent phase (or frequency) envelope. The pulse is frequently characterized by the **pulse duration** (ie, time for which B_1_^+^ is nonzero) and a maximum **B**_**1**_^**+**^ **amplitude** (in μT). The B_1_^+^ amplitude may also be expressed in units of Hertz after division with the gyromagnetic ratio.

Bloch equations or quantum mechanical spin‐system simulations can be used to calculate the frequency‐dependent **pulse profile** B_1_^+^(ν) ** (Figure 2B), the main parameters of which are the **bandwidth** (frequency range over which spins are affected and where minimum/maximum of this range are defined by the FWHM of the pulse profile) and the **flip angle** indicating the extent of rotation of magnetization. Figure 2B shows that the pulse profile and, hence, the effective bandwidth may depend strongly on the applied B_1_^+^ amplitude. While the pulse profile is largely insensitive to B_1_^+^ variation for adiabatic pulses beyond a certain B_1_^+^ threshold, the profile is very sensitive for amplitude‐modulated pulses. Correct calibration of the B_1_^+^ amplitudes is, therefore, critical to achieve the intended pulse profile. **Transmit power adjustment** (**pulse calibration)**, ie, the adjustment of transmit voltage, is performed to adjust the B_1_^+^ amplitude to obtain the intended flip angle. For an ideal frequency selection profile, the magnetization is constant within the bandwidth (excited or inverted) and nonexistent or unaffected outside. However, in practice the pulse profile must be divided into the **pass‐band** (ie, the region where the pulse acts as intended), the **stop‐band** with nominally no response and in between the **transition‐band**. Deviations from the ideal rectangular profile (ie, broad transition‐band or large ripples in the pass‐band or stop‐band) are characteristics of low quality localization (ie, **slice profile imperfections**).

The application of a **localization gradient** (spatially dependent B_0_) simultaneously with the RF pulse translates the frequency‐dependent pulse profile into a spatially dependent **slice profile** (Figure 2C), the main parameter of which is the **slice thickness**, characterized by the FWHM of the slice profile in Hz converted to mm:

$$\text{slice thickness}\;\left[\mathrm{mm}\right]=\frac{\text{pulse bandwidth}\;\left[\mathrm{Hz}\right]}{\text{gyromagnetic ratio}\;\left[\frac{\mathrm{MHz}}{T}\right]\cdot \text{gradient amplitude}\;\left[\frac{\mathrm{mT}}{m}\right]}.$$

Since the pulse bandwidth definition is often fairly inconsistent, VOI volumes can be defined very differently for different vendors. The position of a selected slice can be correctly adjusted only for a single resonance frequency (reference frequency), while it is shifted linearly orthogonal to the slice for off‐resonance frequencies. This shift of the slice profile for resonances at different frequencies is termed **chemical shift displacement error** (CSDE) and is usually expressed by spatial shift in %/ppm, where % refers to the amount of shift expressed as a percentage of the nominal slice thickness. Increasing the pulse bandwidth and gradient strength reduces the CSDE linearly. The practical relevance of the CSDE and its acceptable extent has recently been discussed.^52^

The combination of spatial selection modules in three dimensions defines the location and volume of the VOI. The quality of the volume selection can be expressed by **selection efficiency** (actually selected signal within VOI vs. totally available signal in VOI), **suppression efficiency** (1 minus actually selected signal outside VOI vs. totally available signal outside VOI), and a measure for **signal** **contamination** (total selected signal from outside VOI vs. total selected signal from inside and outside VOI)^48^, ^53^ (Figure 2D). These measures (expressed in ratios or percent) allow for quantitative comparisons of localization performance of different localization schemes, if phantoms with appropriate compartment shape and sizes are used, as suggested elsewhere.^48^, ^53^

Localization performance also relies on the use of **spoiler gradient pulses** (eg, two equally sized gradients encompassing selective refocusing elements) and optional **phase cycling** (ie, changes in relative phase between RF pulses and receiver for different acquisitions).^54^ They can both be applied to prevent contaminating signals from remote areas (from spurious echoes, ie, signal echoes from untargeted coherence pathways), while spoiler gradients reduce contamination from the RF transition‐zone of the refocusing pulses. **Outer volume suppression** (OVS) pulses can suppress signals from all areas, but care must be taken not to create unintended contamination via stimulated echoes created in combination with any of the other applied pulses or to suppress signal from inside the VOI if OVS pulses are not sufficiently broad‐banded to avoid CSDE or are applied to saturate regions very close to the VOI.

### 3.3 MRSI

**FIGURE 3:**
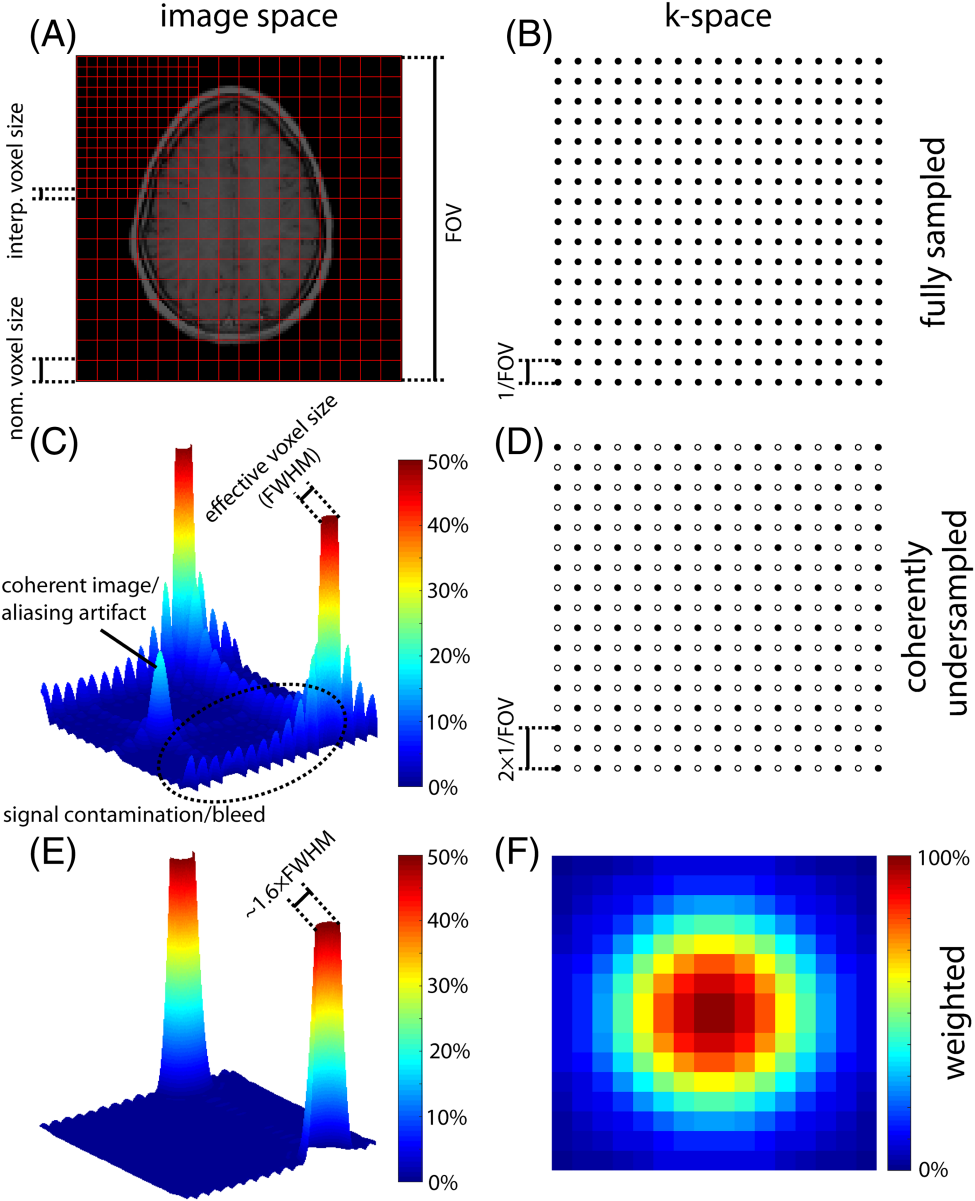
Illustration of terminology for MRSI encoding in image space (left) and k‐space (right). (A) The FOV (exemplified by a slice through a head) is subdivided into a matrix of 16 × 16 voxels with the nominal voxel size defined by FOV/matrix size. The matrix size is determined by (B) the number of k‐space encoding points in each dimension. The size of the FOV is determined by the distance between adjacent k‐space points (1/FOV). Zero‐filling (adding zeroes in the periphery of this acquired k‐space) results in (A) interpolation to smaller voxels in image space (interpolated voxel size). In contrast to acquiring all k‐space points (full sampling in (B)), leaving out k‐space points in a systematic pattern (illustrated by a checkerboard pattern in (D), where empty circles stand for not acquired data) is called coherent undersampling. Such a violation of the Nyquist criterion (ie, the distance between two adjacent k‐space points is >1/object size) causes aliasing artifacts in image domain (C). For coherent undersampling the signal of a point source will therefore not only have contributions from adjacent voxels (ie, signal contamination/bleed caused by discrete Fourier sampling), but also signal contributions from distant voxels through the aliasing artifacts. This is illustrated in (C) via the SRF. The vertical axis (signal amplitude) is cut off at 50% of the maximum of the SRF to illustrate that the effective voxel size is typically assessed as the FWHM. The point source is displayed with a FOV/2‐shift to highlight the signal bleed caused by discrete Fourier sampling. Multiplication of k‐space with a weighting function (Hamming filter illustrated in (F)) reduces the contribution of high‐frequency components in image space. When comparing the SRF in the weighted (E) and nonweighted case (C), it becomes evident that k‐space weighting reduces signal contamination/bleed significantly, but increases the effective voxel size (the spatial response to a point source measured at FWHM)

The spatial extent over which data is acquired in MRSI is specified by the **field of view** (FOV) (Figure 3A). The FOV is the 2D or 3D region that is mapped without spatial aliasing artifacts from violation of the Nyquist criterion after all image reconstruction steps are performed. It corresponds to the inverse of the largest distance of neighboring k‐space points. Often, the FOV is chosen to encompass the entire object within the sensitive coil volume so as to ensure the absence of aliasing. There are several parameters for reporting how the FOV is subdivided into multiple voxels. The **nominal voxel volume**, which is easy to calculate, but tends to mislead in its interpretation, is the FOV divided by the acquisition matrix size, which is the number of encoding samples in each spatial dimension. Image reconstruction commonly includes some form of spatial interpolation, leading to the **interpolated voxel volume**, which is equal to the FOV divided by the final image matrix size. Spatial smoothing (ie, apodization in k‐space) or regularization leads to the **effective voxel volume**, which defines the volume that actually contributes most of the signal at each point and determines the final spatial resolution. The effective voxel volume is defined by the **spatial response function** (SRF), which describes for a single voxel in image space the weighted contribution to its signal from each point in object space. This is in contrast to the **point spread function** (PSF), which describes how a single point in object space propagates across image space. The PSF/SRF can be obtained by numerical evaluation of unit‐intensity k‐space that includes all weightings. Since the SRF is a spatially varying function, the effective voxel volume is often defined as the volume encompassed by a contour determined at FWHM from the top of the SRF. The SRF/PSF reflects the **k‐space weighting**, which can be modified as part of the acquisition (eg, via elliptical or density‐weighted sampling)^55^ or processing (eg, spatial Hamming filtering), and determines the **signal contamination** (also called **signal bleed**), which describes the signal coming in principle from all, but in practice predominantly neighboring, voxels. This contamination differs from that introduced by motion or any other temporal instabilities between acquired k‐space points, which can also originate from remote areas.

**k‐space undersampling**, where the spatial sampling distribution does not meet the Nyquist theorem requirements, is applied in MRSI mainly to reduce acquisition time and is used with a number of image reconstruction methods. This can be done by omitting points in **regular**/repeating **k‐space patterns** (Figure 3D) (leading to **coherent image artifacts**; Figure 3C) or by omitting points in **irregular patterns** (leading to noise‐like **incoherent image artifacts** if not corrected for). Image reconstruction methods that restore missing samples include parallel imaging^56^, ^57^, ^58^, ^59^ or compressed‐sensing reconstruction.^59^, ^60^, ^61^, ^62^, ^63^

**Extended k‐space sampling** indicates that a larger k‐space with unchanged distance between adjacent k‐space points is sampled. This leads to a smaller nominal voxel volume, with an associated loss of SNR, but it can be used to minimize intra‐voxel B_0_ inhomogeneity. With appropriate processing, including B_0_ correction followed by spatial smoothing, the SNR loss can be partially restored while maintaining the improved spectral linewidths.^64^, ^65^

In contrast to traditional **phase encoding** that samples one k‐space point per excitation, **spatial‐spectral encoding** acquires several k‐space points along a **sampling trajectory** simultaneously with the spectral information.^66^ For spatial‐spectral encoding, the SRF for off‐resonance spins may be altered, which is visible as **image blurring** (ie, lowered spatial resolution) or image shifts.

### 3.4 Editing and two‐dimensional MRS

**Spectral editing** (or just “editing”)^67^ aims at selecting target resonances by eliminating all other MR signals that overlap/interfere with the target resonance. **Editing efficiency** compares the signal yield in the final edited spectrum against a spectrum without editing. Different definitions have been used for the reference spectrum. Using the non‐edited spectrum at the same TE introduces a complicated dependence on implementation/application details for the reference spectrum (four‐compartment effect,^68^ specific spectral resolution/shim) and can thus also lead to editing efficiencies of >100%. Hence, it is recommended to conceptually define editing efficiency as the ratio of the integral of absolute values under the edited resonances (with relaxation weighting removed) to the same measure of those resonances in a spectrum acquired with zero TE and an equal number of acquisitions as needed for the edited spectrum. To distinguish between the theoretical limits of an editing method and the editing efficiency for a specific implementation^67^ (in terms of, eg, properties of the applied RF pulses for editing and localization) and experimental situation (eg, shim, field strength), it is proposed to distinguish between a theoretical and a practical editing efficiency. The **theoretical editing efficiency** is to be estimated based on ideal experimental conditions, like optimal shim and ideal RF pulses for editing and localization, which can be derived either from theoretical quantum mechanical calculations (eg, using the product operator formalism^69^) or quantum mechanical spin system evolution simulations. By contrast, the **practical editing efficiency** refers to the situation when the real effects of the actually discussed experiment and properties of the targeted metabolite signals are included and would best be determined with the actual pulse sequence in phantom measurements on single metabolite solutions acquired with realistic B_0_ distribution but long T_2_s (to minimize effects of T_2_ correction).

Both of these editing efficiencies can be distinguished from a broader defined **editing yield**. The editing yield represents signal retention in the edited spectrum after editing (including all signal loss due to the above‐mentioned limiting factors, as well as relaxation) relative to signal obtained under best achievable conditions without editing for the same experimental situation and the full detectable metabolite spectrum. The reference spectrum for the editing yield may therefore refer to the full non‐edited metabolite spectrum with the shortest TE possible, while editing is performed at longer TE with additional RF pulses for coherence selection.

***J*‐difference editing** refers to a class of spectral editing techniques that use subtraction of two sets of scans, with and without *J*‐refocusing to remove unwanted resonances. Thus, these methods require two separate scans (often called the edit‐on and edit‐off experiments). In *J*‐difference editing, such as MEGA‐PRESS (note: **MEGA** originates from the names of its inventors^70^), the editing efficiency refers to the absolute area under the edited (ie, difference) spectrum divided by two times the absolute area under the targeted resonance in a spectrum at TE = 0. The theoretical editing efficiency is 50% in the case of a triplet signal (eg, GABA‐editing with full cancellation of the central line and full recovery of the outer lines) and 100% for a doublet (eg, lactate).

By contrast, **multiple‐quantum‐coherence filtering (MQF)** ^4^, ^67^ is a single‐shot editing method based on the generation (and reconversion to detectable single‐quantum coherence) of multiple‐quantum coherences in *J*‐coupled spin systems and selective observation of signals that pass the coherence filter.

**Co‐editing** refers to all resonances that are detected using a specific editing scheme. It occurs when the frequency‐selective editing pulse is broad enough to excite coupling partners in multiple compounds. As a nuisance, it also refers to contamination due to nonideal frequency‐selective editing pulses. For example, when editing the ^2^CH_2_ signal of GABA at 3.01 ppm by placing an editing pulse at 1.9 ppm, a macromolecule resonance at 2.98 ppm is co‐edited that is coupled to a multiplet at 1.7 ppm because the editing pulse placed at 1.9 ppm is not selective enough. In addition, co‐editing of unwanted signals occurs if the editing pulse saturates untargeted peaks in the edit‐on part only (eg, NAA in GABA editing). **GABA** ^**+**^ is a term used for the sum of signal from GABA, homocarnosine and this inadvertently co‐edited macromolecule signal. Co‐edited resonances should be reported and ideally their contributions estimated. The term co‐editing is also used if multiple metabolite signals are intentionally extracted.^71^, ^72^, ^73^

**Two‐dimensional spectroscopy** (or multi‐dimensional spectroscopy as a more general case with sampling of the temporal spin‐system evolution in multiple periods) can be seen as a form of editing since it can deconvolve overlapping signals into multiple dimensions, thus reducing overall signal overlap.^74^ Obviously, multi‐dimensional MRS goes beyond editing in that the full spectral information is maintained, possibly at the cost of increased measurement time needed and some complexity involved in data modeling.^75^, ^76^, ^77^ In vivo, 2D *J*‐resolved MRS^78^, ^79^, ^80^, ^81^ and variants of the COSY^79^, ^82^, ^83^ method are most popular.^74^, ^84^ For further details, the reader is referred to the literature on high‐resolution NMR.^4^

### 3.5 Magnetization and saturation transfer

**Magnetization (or polarization) transfer** (MT, PT) is generally referred to as the transfer of spin or spin coherence from/or spin coherence from one population of nuclei to another.^85^ Besides general magnetization transfer involving an invisible pool of protons,^86^, ^87^ in MRS this is commonly applied to nuclei exchanging between one chemical species and another (**chemical exchange**, eg, those involved in a chemical reaction,^88^ or between water and labile protons on metabolites^89^), or nuclei of the same compound exchanging between different physical states (eg, free and bound). The technique most often used is **saturation transfer.** ^90^ In this technique, one set of spins is selectively saturated during a certain period of time in which they can exchange with another set of spins, resonating at a different frequency, so that these also become (partly) saturated. The size of this perturbation is then recorded in a difference spectrum, from which exchange rate constants can be derived. **Inversion transfer** is based on the same principle but using selective magnetization inversion rather than saturation.

**Chemical exchange saturation transfer** (CEST) refers to a method for metabolite‐ or macromolecule‐weighted imaging by exchange of labile protons on these metabolites with those on water.^91^, ^92^ Transfer of magnetization can also include indirect (relayed) exchange of polarization by dipolar cross‐relaxation pathways in the form of the nuclear Overhauser enhancement (NOE).^92^

Further terms in the context of MT or PT include **longitudinal relaxation enhancement**, which refers to a technique exploiting the exchange of protons or magnetization from water to metabolites during the recovery period of a MRS experiment to expedite recovery to longitudinal equilibrium magnetization,^93^, ^94^ and **hyperpolarization.** ^95^, ^96^, ^97^ The latter refers to all techniques that allow creating longitudinal polarization above the thermal equilibrium for the specific nucleus. Hyperpolarized ^13^C MRS is presently particularly well established for both animal and clinical research use.^98^, ^99^

## 4 TERMINOLOGY FOR THE DESCRIPTION OF DATA PROCESSING AND QUANTIFICATION

### 4.1 Data processing

The terms **preprocessing** and **processing** are often used interchangeably, but originally, preprocessing referred to data manipulation before FT, while today preprocessing often refers to all operations that are applied to the acquired raw MRS data to prepare it for model fitting^100^ or statistical analysis. In line with the initial terminology, **postprocessing** would refer to data manipulations after FT.

Most of these preprocessing operations fall into one of three categories: (1) operations to reduce the dimensionality of the raw data (coil combination, averaging in the case of multiple recorded scans, and subtraction of sub‐spectra in the case of difference spectroscopy or subtraction‐based localization techniques); (2) operations to remove spectral imperfections (eddy‐current correction [see below], motion correction, frequency and phase‐drift correction, alignment of subtraction sub‐spectra, nuisance peak removal, and baseline correction); and (3) operations to enhance the quality of spectral display (phase correction, apodization, and zero‐filling or signal extrapolation).

#### 4.1.1 Procedures involving the time and frequency domains

**Coil combination:** Combination of data from individual coil elements for the best SNR (and possibly lineshape^101^) using scaled phase‐coherent summation; while in MRSI, spatial deconvolution may be a further aim, if parallel imaging acceleration was invoked.

**Frequency and phase instability correction:** Physiologic and gross subject motion, as well as technical instabilities, lead to scan‐by‐scan variations in signal frequency, phase or amplitude. If single scans have been stored separately, frequency and phase shifts can be corrected retrospectively by matching each acquisition to a reference scan, while the amplitude can only be corrected in special cases.^101^ **Prospective drift correction** refers to correction of the resonance frequency (and possibly also the localization region and shim currents) in real time during the acquisition. These **real‐time adjustments** (see "prospective motion correction" in section 6.0 ) can be calculated from navigator measurements that are interleaved with the MRS/MRSI acquisition or hardware‐based (eg, optical) tracking.

**Zero‐order phase correction:** Adding a constant phase to every point in the complex spectrum (or FID) such that the real and imaginary channels of the spectrum show pure absorption (symmetric positive signal in the case of Lorentzian or Gaussian decaying FIDs) and dispersion character (antisymmetric appearance), respectively.

**First‐order phase correction:** Adding a phase that changes linearly with frequency to the spectrum (equivalent to shifting the TD signal) such that the real and imaginary channels of the spectrum show pure absorption and dispersion character, respectively (with proper zero‐order phasing) for singlet resonances. First‐order phase errors in echo‐based methods should be prevented by precise calibration of the time when the first FID data point is sampled.

**Eddy‐current correction:** Correction of time‐varying phase distortions in the FID caused by eddy currents via multiplication with a time‐varying phase term, often obtained from a non‐water suppressed acquisition with identical gradient scheme.^103^ (If the water signal is not dominating the non‐water suppressed spectrum [eg, in ^1^H MRS of fatty liver or breast tissue], a difference signal obtained from the non‐water‐suppressed minus the water suppressed spectrum may serve as a reference). Strong eddy currents also lead to amplitude distortions (not correctable by eddy‐current correction) that can be eliminated by deconvolution with a reference signal^104^ but may cause noise enhancement. Inclusion of an arbitrary lineshape function or the lineshape function derived from the reference signal in the fitting model may be superior.^20^, ^31^

**Apodization:** Multiplication of the complex‐valued TD signal with a time‐varying weighting function. Most often this is done to attenuate noise towards the end of the FID, while preserving signal at its beginning. This results in a gain in FD SNR at the cost of spectral resolution and invalidates the concept of TD SNR (time‐varying noise). Apodization (in the original meaning of the term) can also be applied to achieve a smooth transition to zero amplitude at the end of the recorded FID to prevent the Gibbs ringing artifact (also called truncation artifact) associated with zero‐filling of non‐decayed signals. Apodization in TD should not be applied prior to model fitting because it alters the noise characteristics. Apodization in spatial direction does not incur this fitting problem.

**Lineshape transformation:** Transforming the lineshape in the recorded data to a target lineshape, either correcting lineshape imperfections^104^, ^105^, ^106^ or aiming at **resolution enhancement** (Lorentzian to Gaussian transformation). It involves deconvolution by the original lineshape function and convolution with the target function, both of which are applied in the TD and can be understood as another form of apodization.

**Digital filtering:** Mathematical operations, usually on the FID, to either alter the spectral width or affect signals in specific frequency bands. Digital filters are also in use in the k‐space domains for MRSI data.

**Elimination of spurious signals:** Preprocessing methods can be applied to remove residual water and lipid signals. Convolution‐difference filtering^107^ and **singular value decomposition** (SVD, where the Hankel–Lanczos implementation, HLSVD, is particularly efficient)^108^ have been widely used for water removal. SVD methods represent the signal as a sum of exponentially decaying sinusoids and then subtract signals that fall in a specific frequency band. For elimination of spurious lipid signals in MRSI, see section "Lipid signal reduction" in section 4.1.2. "Procedures involving the spatial domains".

**Zero‐filling:** Adding zeroes to the end of the FID prior to FT corresponds to interpolation of data points in the FD, increasing the digital resolution in the FD. (Note: “zero‐padding” is an equivalent term but is uncommon and not recommended). Zero‐filling by a factor of 2 also augments the information content in single‐channel spectra (either absorption or dispersion) and is thus helpful for peak integration or model fitting restricted to the FD absorption channel,^109^, ^110^ but adds no additional information for fitting of FIDs or complex‐valued spectra. †† Zero‐filling by at least a factor of 2 is recommended to improve visual representation of spectra^109^ unless it introduces truncation/ringing artifacts (see above, under "Apodization"). Zero‐filling is counter‐indicated before model fitting, unless the model basis spectra have been treated equivalently or all signal amplitudes have decayed to zero at the end of the recorded FID.

**Baseline correction:** Applying algorithms that model and subsequently subtract the baseline by invoking mathematical base functions that usually do not have a physical meaning but just represent the broad features of the baseline (eg, by splines,^20^ wavelets^111^) or the initial part of the FID.^112^ It is recommended to apply these procedures simultaneously with signal modeling as opposed to a preprocessing step. This amounts to semi‐parametric estimation,^32^ which entails setting of subjective **hyperparameters**.

**Frequency correction** either refers to frequency alignment of multiple spectra (see above, under, "Frequency and phase instability correction") or to setting the chemical shift scale (see section 2.2).

#### 4.1.2 Procedures involving the spatial domains

**Lipid signal reduction:** MRSI studies often avoid including extracranial lipids in the preselected VOI. Studies that aim to sample a wide FOV that includes extracranial lipid regions typically need to apply processing or reconstruction methods that reduce lipid signal contamination. Specific methods include lipid k‐space interpolation^113^ and constrained reconstruction.^114^, ^115^

**Spatial registration:** is applied to register the location of a single voxel or the MRSI grid volume elements (or the “grid”) to MR images, either commonly recorded images or segmentation maps, to assign partial volumes of white matter (WM), gray matter (GM) and CSF.

**MRSI reconstruction** refers to a series of steps that transform the raw data to a final grid of spectra (similar to MRI).^116^ This can include the process of bringing all k‐space data to a Cartesian coordinate system (**regridding**), the combination of signal from different receive coil elements (**coil combination**), recovery of omitted k‐space points (via **parallel imaging** or **compressed‐sensing** reconstruction), transformation from k‐space to image‐space, modification of the SRF by applying a **spatial filter**/**k‐space weighting**/**k‐space apodization**, and finally **spatial interpolation** (eg, zero‐filling) or alternatively shifting the reconstructed matrix by a user‐determined fraction of the nominal voxel size (**grid shifting**) to reduce partial volume effects.

### 4.2 Quantification procedure

Conversion of the processed MRS signal into quantitative results involves two parts: first, data analysis (ie, determination of numerical intensity results for the spectral features in arbitrary units, usually by parameter estimation ‡‡ from a model fit), and second, **quantification** (also called **quantitation**) (ie, conversion of numerical data to reportable units).^100^

#### 4.2.1 Area and parameter estimation

**Peak integration:** Determination of peak area by digital peak integration is the simplest way towards signal intensity estimation, but can only be used for well‐isolated peaks and is affected by baseline offsets.

**Least squares model fitting:** Model fitting algorithms have been developed to find those model parameters that minimize a **cost function** representing the deviation of the modeled signal from the measured signal. In the case of least squares fitting, the cost function (often called χ^2^ in this case) is constructed as the summed squared difference between the model and the measurement calculated over the full or a limited data range in TD or FD. Fitting routines in MRS are often categorized as **TD** or **FD fitting methods**. This differentiation is based on the domain in which the cost function is calculated and not on whether the signal model is formulated in TD or FD. Most fit packages calculate χ^2^ for complex‐valued data, ie, they make use of real and imaginary data channels to be essentially independent of proper zero‐order phasing and to make use of the complete signal information independent of zero‐filling (see above). If the model signal is expressed as the sum of analytical functions in FD, experimental deviations from the ideal model cannot easily be included (eg, initial point bias, or signal truncation), and must be dealt with in preprocessing. Assuming that the noise has a Gaussian probability density function, least squares fitting can also be regarded as **maximum likelihood parameter estimation.** ^117^

**Prior knowledge or a priori information:** This refers to information that is known and is fixed in the signal model or used as constraints on the model parameters. The use of prior knowledge helps to: (1) facilitate finding the global minimum of the cost function; (2) direct the fitting process towards interpretable results; (3) prevent physically meaningless outcomes; and (4) increase the fitting precision. Usually three types of prior knowledge are enforced:

- Composition of the tissue and the spectrum: instead of using multiple independent resonance signals, the fit model is set up (in part) as a linear combination of known compound responses (obtained from equivalent in vitro measurements or in silico simulations) of known contributing metabolites. This type of fitting is generally termed **linear combination model fitting** (eg, LCModel^20^). §§
- Relations between fit parameters: relations between fit parameters are enforced to reduce the number of unknown parameters, either because they are given by first principles or are expected based on the literature. Each relationship reduces the number of parameters to be fitted and thus reduces the variance in the estimated parameters (ie, the Cramér‐Rao minimum variance bounds; see below). Usual relations include predefined ratios for signal areas and additive relations for frequencies, phases and **relaxation rates** (inverse of relaxation time constants).
  As an example of a basic‐principle‐based relation, the zero‐order phase of all resonance lines is usually known to be identical to a very good approximation and is thus enforced. Expectation‐based relations often concern the relaxation rates, where they are assumed to be equal between metabolites or between different resonance patterns within a metabolite

- Restriction of parameter space: the allowed parameter space is restricted to a physically meaningful range (eg, positive amplitudes, ie, concentrations, and decay rates) or to an assumption‐based range (eg, T_2_ values to remain in a range that is known to be reasonable from earlier work).

**Quality of model fitting:** To judge how well the signal model at the final minimum of the cost function agrees with the recorded data, χ^2^ can be put in relation to what is expected for the case that the **fit residuals** (signal model minus experimental signal) originated from pure measurement noise. This ratio is called the **fit quality number** (FQN). ¶¶ If FQN > 1.0, the obtained model does not fully match the experimental signal (eg, indicating a local, and not the global, minimum of the cost function, unaccounted for artefacts, differences in lineshape, non‐included resonance lines, ill‐described baseline or water residues). For FQN = 1.0, the model perfectly agrees on average with the data within the precision allowed by the noise. By contrast, for FQN < 1.0, the model over‐fits the data, ie, the model appears to have too much flexibility ## for the given data (or the noise was overestimated).^118^ A FQN of about unity does not prove the accuracy of the model or the obtained parameters; it is only a measure to judge agreement between the data and the assumed model. It is recommended that fit packages provide FQN values or equivalent metrics to allow judging the match of the model used in MRS evaluations.

#### 4.2.2 Error estimation

Since a good‐looking fit does not guarantee that the fit parameters are reliable, the **uncertainty of the estimated parameters (fit error)** must be computed and reported (at least for the relevant parameters, most often the peak areas as proxies for metabolite tissue contents). A good error estimate is provided by the so‐called **Cramér‐Rao minimum variance bounds** (CRMVB),^117^, ^119^ more commonly referred to as **Cramér‐Rao lower bounds** (CRLB) but also abbreviated as CRB (not recommended). They represent the minimal possible theoretical uncertainties given the estimated model parameters and the size of the noise. There are widespread misconceptions regarding the correct interpretation of these errors: they neither reflect the appropriateness of the model, nor the quality of the fit. Additionally, it is important to understand that this error is only valid under the assumptions made: (1) the assumed data model is valid, (2) the estimated parameters are nearly correct (FQN~1; see above), and (3) that the noise is Gaussian. The CRLB do not depend on whether the least squares fit is performed in TD or FD, if the same TD model is applied and the full range of TD and FD data is used. It should also be realized that the definition of CRLB leads to error estimates in the same units as the parameter itself, not fractions of the estimated parameter expressible as percent values. In this context, it should be noted that the use of relative error estimates as a quality index to eliminate single metabolite estimates can readily lead to biased cohort data or elimination of relevant findings.^120^, ^121^ CRLB for quantification of metabolites with similar strongly overlapping spectral patterns are intrinsically high. Therefore, combined pools of metabolites are often evaluated, rather than their components (eg, tNAA = NAA + NAAG, tCr = Cr + PCr, tCho = PCh + GPC, Glx = glutamate + glutamine).

An alternative approach to estimation of fit error that includes both uncertainties from interdependence of model parameters (like for CRLB) and deviations of measured data from fitted model (like in FQN) was presented in terms of **confidence limits**.^122^

#### 4.2.3 Quantification (quantitation) step

This represents the conversion of machine‐dependent units for metabolite **tissue concentration** to reportable ones.^100^ The term **absolute quantification** was originally coined for the conversion to standard concentration units, where the conversion includes all corrections needed based on measurements and calibrations for the currently reported case^123^ to contrast with relative quantification. However, this narrow meaning is an idealization that normally cannot be realized in practice and “absolute quantitation” today is also used for plain conversion to standard units with calibrations from earlier studies or literature values, but this usage is not encouraged (see below).

**Units** (for details on quantification and concentration units see reference^100^ in this issue, reference^123^ and references therein): the most common form of scaling is to express results in terms of unitless **ratios with regard to other metabolites or water**. If this is done, it should be made clear whether the ratios stand for relative concentration ratios (derived from fitting the whole spectral pattern of both quantities and potentially including relaxation time corrections) or peak area ratios (of possibly the singlet part of the metabolite spectrum and not accounting for the number of protons responsible for a resonance). Equally, if using ratios to the unsuppressed water signal, it must be clear whether they are area or concentration ratios. Frequently, the reference to water is further calibrated and results are reported in **absolute** chemical **units**. Care has to be used to differentiate between **moles per mass of tissue** (mol/kg), **moles per volume of tissue** (mol/L, **molar, M**), and **moles per mass of solvent** (tissue water) (mol/kg solvent, **molal, m**). Molal units are the most straightforward measure to derive from MRS with unsuppressed water as the reference and closest to the chemically relevant intracellular concentrations, but they have not been used much. For the other units, water content and possibly density of tissue must be measured (eg, using signal from pure CSF) or assumed. If assumptions are involved (with respect to tissue content of a reference substance [metabolite or water], volume fractions or any other relevant tissue properties [eg, longitudinal (T_1_) or transverse (T_2_) relaxation times of water or metabolite resonances] ) that can substantially alter the reported values if not correct, it is preferable to use a term like “determination of tissue concentrations in absolute or institutional units based on the listed assumptions” rather than the term “absolute quantitation”.

If not all corrections needed for the conversion to standard units are applied or if the conversion relies on local acquisition specifics or non‐verified assumptions, estimated concentrations are often expressed in so‐called **institutional units**. They are meant to provide units allowing for detection of alterations in tissue concentrations when using the same local methodology. However, they do not necessarily provide the basis for comparison with results from other sites or involving other methods.

Absolute quantification methods also include referencing via external calibrations using **external standards** (vials with solutions of known content) or RF signals.^123^, ^124^ Both methods include specific (neither trivial nor necessarily robust) ways to correct for B_1_^+^ and B_1_^−^ inhomogeneities and possibly further calibration steps.

In voxels with mixed tissue and CSF contributions, **compartment effects** (differential signal contributions from CSF, GM and WM) need to be taken into account. No consistent terminology can be used as a shortcut around explicit explanations. In addition, **relaxation corrections** (for metabolites and reference compound) have to be either reported in detail (approximate or exact formulas; differentiation of relaxation times per metabolite or per resonance), or, if they were left out, this should also be mentioned.

**Partial volume contributions:** In vivo measurements rarely originate from a single tissue type, and the **relative tissue volume fractions** represent the partial volume contributions (see above under compartment effects). One important situation where this needs to be accounted for is in the reporting of single metabolite concentration values (but not for metabolite ratios) where there is some volume contribution from materials that contain no observable metabolites, such as air or bone.

**MR visibility:** Metabolites that are subject to considerably faster transverse relaxation than expected (or simultaneously detected signals) or other processes affecting signal yield (eg, chemical exchange) yield low MR signals. This is often referred to as reduced MR visibility. This can be due to specific effects of a micro‐compartment (eg, if bound to a macromolecule), meso‐compartment (myelin layers), or the substance as such (phospholipids). The effects of differential MR visibility are also particularly apparent with quadrupolar nuclei such as ^23^Na or ^39^K.

### 4.3 Sources and characterization of variance in estimated results

Basic terminology with regard to measurement errors for MRS is in principle no different to that of any other quantitative measurement in the medical sciences (eg, reference^125^), but the exact terminology for repeated scans or multi‐center studies is of interest.

**Agreement versus reliability:** Agreement is determined from the results of the compared measurements themselves, not in relation to variance of the measured parameter between subjects or with regard to other variable factors. Reliability, on the other hand, puts agreement in relation to variance in cohorts or other variable settings. Agreement can be expressed using Bland–Altman plots and statistics (including **test–retest coefficients of variance** [CV]), while reliability is reflected in terms like intraclass correlation coefficients.

**Accuracy** refers to the agreement between measured/estimated and ground truth values (eg, metabolite tissue contents). However, since the ground truth tissue content is rarely known, accuracy is usually not (and mostly cannot be) reported in MRS. For **precision**, see the discussions on repeatability (below) and CRLB (in section 4.2.2).

**Repeatability versus reproducibility:** The term repeatability is used if measurements are repeated in the same setting with the same operator and under identical conditions, while reproducibility refers to repeated measurements under different settings, eg, another method (methods comparison), operator (inter‐observer variance), another instrument (scanner or manufacturer dependence), site, or B_0_.

**Sources for the observed variance** for quantified metabolite concentrations or ratios can be classified as technical or biological. Cohort differences or within‐person changes in metabolite levels are only detectable if they are larger than the uncertainty from the technical variance, which should be determined by repeatability studies when presenting new measurement or evaluation methods. Technical factors that lead to variability in quantitative MRS results within the same scanning session and without re‐prescribing the acquisition include spectral quality (as defined by SNR, linewidth, appearance of artifacts) and the stability of the hardware (and also the human subject in terms of gross or physiologic motion). This is captured by the **scan‐to‐scan repeatability** of an MRS exam. Between scanning sessions, additional factors affect the **short‐term reproducibility** of measurements, including intermediate‐term stability of the hardware and stability of prescan results (eg, B_1_, B_0_ optimization), but also subject placement (including table position vs. isocenter) in the scanner (eg, relative to coil or magnet) and placement of the VOI.^126^ If a whole exam is repeated in full, which means by the same operator at the same site and with the same scanner while physiologic changes and learning/memory effects can be excluded, one could still speak of **exam repeatability**. (In particular, this would also imply that any knowledge from the previous prescription should not be used, eg, no screen shots of the previously prescribed VOI). In multi‐site trials, variability in the pulse sequence and parameters, the calibration steps prior to MRS data acquisition, any hardware differences and processing and spectral fitting methodology may introduce additional variance in quantitative MRS results.^127^ Beyond these technical factors, biological factors including diurnal, day‐to‐day, diet‐ or stimulation‐related physiological changes and **inter‐subject** (including age, gender, ethnicity)^128^ **differences** can introduce variance in MRS data. For example, in clinical trials, time of day and time since last drug dose may be critical factors.

Which particular evaluation of variance is relevant in a particular study depends on the biological question at hand.^126^, ^127^, ^130^, ^131^, ^132^, ^133^, ^134^, ^135^, ^136^, ^137^ For example, repeatability needs to be evaluated in studies that aim to detect within‐session stimulation or drug effects, while between‐session reproducibility needs to be determined when planning a clinical trial lasting for days or weeks and where metabolites are quantified before and after administering a treatment. Detectability of cohort differences depends on within‐cohort variance, which may be different between patient and control groups.

In the absence of repeat measurements, ie, when clinical decisions need to be made based on a single spectrum, CRLB are sometimes used as indicators for a lower estimate of repeatability or even reproducibility, but it should be clear that they are not valid replacements for estimates from repeatability or reproducibility studies.

## 5 TERMINOLOGY FOR THE DESCRIPTION OF MR SCANNER HARDWARE AND ITS CALIBRATION

### 5.1 Hardware

**Magnetic field strength:** **B**_**0**_ represents the magnetic flux density (also, induction of the magnetic field), and is characterized using tesla (T) as the unit. At present, clinical scanners are often categorized according to their **static magnetic field** strength as **low field** for 1.5 T and below, **high field** for 3 T, and **ultrahigh field** for 7 T and above. Note that a 3T scanner may actually only be 2.9 T in reality.

**Gradient performance** is specified by two main characteristics, maximum gradient strength in mT/m (defined per single axis, although higher overall values can be achieved when applying gradients along more than one axis simultaneously) and gradient slew rate in mT/m/ms. Alternatively, gradient rise time (in ms to full amplitude) can be provided.

**B_0_ shim systems** are designed to optimize the B_0_ homogeneity within a target (tissue) volume and can be distinguished according to the maximum degree n *** of the spherical harmonic terms they are designed to compensate: first degree (only three linear gradients), second degree (an additional five terms of the spherical harmonics expansion), third degree (an additional seven terms). Characterization of shim systems should also include the maximum shim strength (in μT/mm^n^) available for each shim element.

**Transmit and receive RF coils:** Transmit coils should be characterized by the number of elements and the maximum B_1_^+^ field (**peak B**_**1**_^**+**^, in μT) that can be achieved in typical settings (average coil load). The peak B_1_^+^ that is supported by the coil can be very important for MRS as it directly limits the maximum achievable bandwidth (inverse to minimum duration of localization pulses). Shorter pulses with higher B_1_^+^ translate into lower CSDE and shorter minimum TE. Receive coils are characterized by the number of independent channels and geometric configuration, and are important determinants of SNR.

### 5.2 Calibration procedures

**Prescan:** Calibrations before each scan are often called prescan procedures. They are usually automated—but can be replaced or extended by optimized tailored sequences—and include **transmit power adjustment** to calibrate the desired flip angles (locally at the VOI rather than on a slice, as often implemented by vendors), **frequency adjustment** (determination of the carrier frequency using the water peak as a reference in ^1^H MRS), **shimming** (optimization of B_0_ shim currents to minimize B_0_ variation in the VOI), adjustment of **water saturation parameters** (most often flip angles) and possibly adaptation of **receiver gain** (analog and/or digital amplification of received signal before digitization). Where applicable, the recording of coil sensitivity characteristics, eg, for coil combination or processing when using parallel imaging methods, is also part of prescan procedures.

## 6 FURTHER GENERAL TERMINOLOGY

**Chemical shift imaging and MRSI:** These terms have been used synonymously and refer to methods that create maps of individual spectral components or metabolite distributions. The term spectral imaging is not used in this context, but rather refers to techniques outside the field of MR. We recommend using MRSI rather than the somewhat historic term chemical shift imaging (CSI). In addition, the terms “chemical shift imaging” and “spectroscopic imaging” have also partially been adopted by the MRI community to represent MRI methods that provide selection of different chemical shift species (primarily fat and water) by the recording of multiple gradient echoes with different echo times to essentially reconstruct density images for different chemical shift species based on in‐phase and out‐of‐phase images of the species under consideration. Even the MESH term “chemical shift imaging” often leads to these Dixon‐based techniques rather than to papers based on MRSI, while “spectroscopic imaging” appeared early on already in the title of Dixon's original publication.^138^ To prevent confusion, we strongly advise calling those methods (ie, [multi‐point] Dixon techniques or, eg, IDEAL^139^) **chemical shift encoded MRI** ^140^ or Dixon techniques.

**Acquisition, shot, trace, single scan, single average, transient, excitation, repetition, single recording:** All these terms have been used in the literature to refer to a single application of the pulse sequence for one recording of data, separated from the next by a delay for partial T_1_ recovery and with the concept that multiple scans are averaged to form a dataset for analysis. In addition, the underlying concept implies that the multiple scans are essentially identical. Possible differences may be due to T_1_ saturation effects, the relative phases of the RF pulses in the acquisition and detection sequence (due to phase cycling), and alterations in RF pulses that are the basis for multi‐shot acquisition sequences for localization (eg, SPECIAL) or editing. Acquisitions may comprise multiple echoes. **Number of averages** (NAV) and **number of excitations** (NEX) are terms to indicate how many single scans were recorded for the averaged spectrum. FID should not be used in this context as it has a narrower meaning. Strictly speaking, repetition should not be used if the recordings differ somewhat, eg, to form a phase cycle or in a multi‐shot localization sequence like ISIS. “Average” and “repetition” result in oxymorons if used with the adjective single. Strictly speaking, “scan” and “shot” are most pertinent if referring to the whole pulse sequence including the excitation and data acquisition (recording of transients) parts. However, “scan” is ambiguous, since the term is also often used to refer a complete set of “shots” comprising a dataset, or an entire scanning session (an MR session).

**Center frequency:** In prescan procedures, the proper frequencies for excitation and reception are set, usually by reference to the largest excited resonance (most often water or methylene peak of lipids). A peak that shows the same resonance frequency as the excitation frequency is said to be **on resonance**; the base frequency at which RF is transmitted is called the **carrier frequency**. On clinical MR systems, this frequency can nowadays often be selected to be in the center of the spectral range of interest (and should be reported in publications as offset from water or as ppm value), while the center frequency for data acquisition normally remains on resonance with the water peak.

**Homonuclear:** Concerning interactions between identical NMR active isotopes (eg, ^1^H‐^1^H).

**Heteronuclear:** Concerning interactions between two different NMR active nuclei (eg, ^1^H‐^13^C).

**Heteronuclear decoupling:** Application of RF pulse schemes to suppress heteronuclear coupling evolution.

**Natural abundance:** Fraction of the relevant, NMR detectable nucleus relative to the total sum of all isotopes, as occurring in a biological sample (eg, the natural abundance of ^13^C is 1.11%).

**Isotope delivery studies:** Investigation of metabolism based on administration of substrates labeled with NMR‐visible isotopes that are of low natural abundance. In the case of intravenous infusions as the mode of delivery, they are often named **isotope infusion studies**.

**Background signal:** In the context of isotope delivery studies, the fraction of the signal originating from the moiety of the NMR‐sensitive nucleus occurring due to its natural abundance.

**Residual dipolar coupling:** Partially detectable dipolar coupling in partially oriented molecules, where the physics is identical to dipolar coupling, but the size of the coupling is reduced by partial averaging reflecting the degree of orientation (eg, in liquid crystals or in vivo in ^1^H MRS of skeletal muscle).^141^, ^142^

**Orientation dependence:** Dependence of a parameter or spectrum on the orientation of the investigated object (eg, muscle fiber or microscopic non‐spherical object) with respect to B_0_.^141^, ^142^

**Susceptibility shifts:** Shift of resonance frequency due to local susceptibility effects either mediated through the susceptibility difference of investigated material or neighboring structure of different magnetic susceptibility (eg, susceptibility shifts of fat or blood vessels vs. tissue). This can lead to peak splitting if a certain metabolite is present in two environments with different susceptibilities (as is the case for intra‐ and extramyocellular lipids^142^, ^143^).

**Functional MRS:** This expression is used in a broad sense as representing MRS performed to reflect any physiologic function (eg, muscle or other organ function) or in a narrow sense referring to brain function in analogy to functional MRI.

**Physiologic motion:** Term usually referring to involuntary movements of whole or parts of organs from motion in connection with physiology, ie, cardiac, respiratory, or peristaltic motion.

**Prospective motion correction:** Term used to differentiate approaches that track body motion and update scanning parameters (VOI coordinates, center frequency and shim values) in real time during acquisition as opposed to postprocessing methods that are used to correct for shot‐to‐shot differences in frequency and phase.

**Recovery time constant:** In the context of functional MRS (eg, ^31^P MRS of muscle after exercise^144^), inverse of the **recovery rate** reflecting the time constant for a mono‐exponential recovery of a metabolite signal after stimulation.

**Triggering:** Synchronization of data acquisition with physiologic motion or other external events. In the case of MRS, it is most often used in extra‐cranial applications (eg., MRS in heart,^145^ muscle, liver, spinal cord^146^), but is also used in brain in the context of diffusion‐weighted MRS.^147^

**Preclinical MRS:** Term referring to in vivo MRS in animals, mostly to investigate human disease models in animals, such as rodents. In addition to the biological factors influencing reproducibility and repeatability as mentioned above for human studies, most preclinical studies also imply the use of anesthetics to decrease the stress (and potential pain in the case of surgical intervention) for animals and to restrict biological motion. The choice of anesthetics, the anesthesia protocol (dose and duration) prior to and during the MRS scan, as well as the way the animal's physiological parameters were monitored and used to regulate the anesthesia, should always be clearly reported, as they might have an impact not only on the reproducibility of the MRS measurement, but also on its repeatability within a scanning session. For a detailed discussion of those aspects, please refer to the dedicated paper on this subject in this issue of *NMR Biomed.* ^148^

## 7 CONCLUSIONS

This report explains terms that describe context, concepts and terminology relevant for in vivo MR spectroscopy, in particular for the description of MRS methods and the properties of in vivo MR spectra. This has emerged as a consensus among a large group of contributing authors who are experts in the field, as documented by their publication histories. It is meant to provide a framework for the other articles in this special issue and to serve as a reference for publications of research in MRS, but also clinical applications where MRS results are mentioned. A summary of the main recommendations on terminology and concepts in in vivo MRS is listed in Table 1, but many other suggestions are to be found in the main text. For ease of reference, Table 2 collects all the terms which have been explained in the text, while Table 3 lists abbreviations for MRS‐specific pulse sequences and software that are sometimes used in the MRS literature in a stand‐alone fashion without either explanation or reference.

## FUNDING INFORMATION

The preparation of this manuscript was in part supported by the Swiss National Science Foundation (320030–175984, RK; 310030‐173222/1, CC); the National Institute of Neurological Disorders and Stroke (NINDS) (grant R01 NS080816, GÖ), the National Institute of Health (NIH) (R01EB016064, AAM), and the Austrian Science Fund (FWF) (projects I 1743‐B13, MM and P 30701‐B27, WB). In addition, the Center for Magnetic Resonance (University of Minnesota) Research is supported by the National Institute of Biomedical Imaging and Bioengineering (NIBIB) grant P41 EB015894 and the Institutional Center Cores for Advanced Neuroimaging award P30 NS076408.

## Footnotes

* The descriptor “nuclear” was dropped for clinical use of NMR to prevent confusion with nuclear medicine methods involving unstable isotopes and high energy irradiation, even although almost all clinical and preclinical use of MR refers to NMR rather than electron spin resonance (ESR).

† For example, Topspin software for NMR by Bruker, LCModel program (although, inconsistent with the current suggestions, LC Model also uses the SD over the full range of the fitting residuals in FD as “noise”). Even a recent consensus paper from this community still tended towards keeping the old‐fashioned NMR convention of two SD.^33^

‡ In metabolite phantoms, 4,4‐dimethyl‐4‐silapentane‐1‐sulfonic acid (DSS) or trimethylsilyl propionate (TMSP) should be used instead, because TMS is not soluble in water.

§ The natural linewidth includes effects of exchange, which are particularly relevant for water.

¶ Microscopic susceptibility variation adds further exponential decay and possibly also Gaussian damping, where local susceptibility regions sampled by a diffusing particle provide mostly exponential decay, while inclusion of multiple non‐exchanging compartments (eg, white matter bundles of different directions) may provide Gaussian character.

# Beware, some manufacturers use the abbreviation NP to stand for the sum of the number of real and imaginary points (Varian, Agilent).

** Where ν represents a frequency (cycles per second) axis, rather than an angular frequency (radians/second) axis, for which the symbol ω is customary.

†† And also for single‐channel data in the case of symmetric echo time domain data.^110^

‡‡ It should be noted that the terms estimate/estimation are used exclusively with their meaning in engineering and statistics, where “parameter estimation is the computation of numerical values for the parameters [for a model representing the data] from the available observations”.^117^ “Estimating” in this technical sense is by no means to be associated with the colloquial meaning of guessing or approximating.

§§ In more detail, for this type of linear combination model fitting,^20^, ^31^ Equation 1 is essentially transformed into the following form:
$S\left(t\right)=\sum_{k=1}^{Q}P{\left(t\right)}_{k}\exp\left(-i\omega_{k}t+i\phi_{k}-\frac{t}{\;T_{2,k}^{*}}-\frac{t^{2}}{T_{G,k}^{2}}\right)+\varepsilon \left(t\right)$, where the summation over L individual resonance signals with individual amplitudes A_j_ in Equation 1 is replaced by summation over Q metabolites with individual signal patterns P(t)_k_ that correspond to the ideal responses of a whole metabolite spin system to a particular pulse sequence as obtained from quantum‐mechanical simulation or high‐resolution in vitro measurement. P(t)_k_ describe the response of metabolite k for standard conditions with minimal or zero linewidth and no offset in frequency or phase. Upon multiplication in TD with a common width, phase and frequency term—just like in Equation 1 for single resonances—the signals matching the in vivo condition to be used as base components for the fit procedure are obtained.

¶¶ In other words, FQN is calculated as the ratio of the variance in the fit residual (in the fitted frequency or time range) divided by the variance for pure spectral noise.

## This is particularly a problem in semi‐parametric modeling where hyperparameters that have no physical reality may be set heuristically and may (also in the case that FQN ≥ 1) erroneously suggest a reasonable fit.

*** Often inaccurately called order instead of degree.

## APPENDIX A Members of the Experts' Working Group on Terminology for MR spectroscopy

**Table:**

Name		Institution
Ovidiu C.	Andronesi	Martinos Center for Biomedical Imaging, Department of Radiology, Massachusetts General Hospital, Harvard Medical School, Boston, Massachusetts, USA
Carles	Arus	Departament de Bioquímica i Biologia Molecular, Unitat de Bioquímica de Biociències, Universitat Autònoma de Barcelona (UAB), Cerdanyola del Vallès, Spain
Adrianus J.	Bakermans	Department of Radiology and Nuclear Medicine, Amsterdam University Medical Centers, University of Amsterdam, Amsterdam, Netherlands
Chris	Boesch	Departments of Radiology and Biomolecular Research, University Bern, CH
Francesca	Branzoli	Institut du Cerveau et de la Moelle épinère – ICM, Centre de NeuroImagerie de Recherche ‐ CENIR, Paris, France
Kevin	Brindle	Department of Biochemistry, University of Cambridge, Cambridge, UK
Patrick	Cozzone	Singapore Bioimaging Consortium (SBIC), 11 Biopolis Way, #02‐02 Helios, Singapore 138667
Dinesh K.	Deelchand	Center for Magnetic Resonance Research and Department of Radiology, University of Minnesota, Minneapolis, MN, USA
Wim	Derave	Dept. of Movement and Sports Sciences, Ghent University, Ghent, Belgium
Wolfgang	Dreher	University of Bremen, Department of Chemistry, Bremen, Germany
Ulrike	Dydak	School of Health Sciences, Purdue University, West Lafayette, IN, USA
Richard	Edden	Russell H. Morgan Department of Radiology and Radiological Science, The Johns Hopkins University School of Medicine, MD, USA.
Uzay	Emir	School of Health Sciences, Purdue University, West Lafayette, IN, USA
Gabriele	Ende	Department for Neuroimaging, Central Institute of Mental Health, Medical Faculty Mannheim, Heidelberg University, Mannheim, Germany.
Geoffrey	Payne	Department of Medical Physics, University Hospital Southampton, UK
Danielle	Graveron‐Demilly	Université Claude Bernard, Lyon 1, Villeurbanne, France
Stephan	Gruber	High‐field MR Center, Biomedical Imaging and Image‐guided Therapy, Medical University of Vienna, Vienna, Austria.
Ashley	Harris	Department of Radiology, University of Calgary, Calgary, Canada
Anke	Henning	High‐Field MR Center, Max Planck Institute for Biological Cybernetics, Tübingen, Germany AND Advanced Imaging Research Center, University of Texas Southwestern Medical Center, Dallas, Texas, USA
Pierre‐Gilles	Henry	Center for Magnetic Resonance Research and Department of Radiology, University of, Minnesota, Minneapolis, MN, USA
Ralph	Hurd	Stanford Radiological Sciences Lab, Stanford, California, USA
Fahmeed	Hyder	Department of Radiology & Biomedical Imaging, Yale University, New Haven, Connecticut, USA
Christoph	Juchem	Departments of Biomedical Engineering and Radiology, Columbia University in the City of New York, USA
Margarida	Julià‐Sapé	Centro de Investigación Biomédica en Red (CIBER), Spain
Hermien E.	Kan	C.J. Gorter Center for High Field MRI, Department of Radiology, Leiden University Medical Center, Leiden, the Netherlands.
Graham J.	Kemp	Department of Musculoskeletal Biology and Liverpool Magnetic Resonance Imaging Centre (LiMRIC), University of Liverpool, Liverpool, UK
Dennis W.J.	Klomp	University Medical Center Utrecht, Utrecht, Netherlands
Jack	Knight‐Scott	Department of Radiology, Children's Healthcare of Atlanta, Atlanta, Georgia, USA.
Fan	Lam	Department of Bioengineering and Beckman Institute for Advanced Science and Technology, University of Illinois at Urbana‐Champaign
Karl	Landheer	Biomedical Engineering, Columbia University Foundation School of Engineering and Applied Science, New York, New York, USA
Hongxia	Lei	Centre d'Imagerie Biomedicale (CIBM), Ecole Polytechnique Fédérale de (EPFL), Lausanne, Switzerland
Yan	Li	Department of Radiology and Biomedical Imaging, University of California, San Francisco, CA, USA
Zhi‐Pei	Liang	Beckman Institute for Advanced Science and Technology, University of Illinois at Urbana‐Champaign, USA
Peter	Lundberg	Radiology and Center for Medical Imaging and Visualization (CMIV),University of Linköping and University Hospital of Linköping, Sweden
Chao	Ma	Gordon Center for Medical Imaging, Department of Radiology, Massachusetts General Hospital, Harvard Medical School, Boston, MA, USA
Erin	MacMillan	The University of British Columbia, Simon Fraser University, and Philips Canada
Craig	Malloy	Advanced Imaging Research Center, University of Texas Southwestern Medical Center, Dallas, TX, USA
Silvia	Mangia	Center for Magnetic Resonance Research and Department of Radiology, University of Minnesota, Minneapolis, MN, USA
Malgorzata	Marjanska	Center for Magnetic Resonance Research and Department of Radiology, University of Minnesota, Minneapolis, MN, USA
Dieter	Meyerhoff	School of Medicine, University of California San Francisco, Veterans Administration Medical Center San Francisco, USA
Mark	Mikkelsen	Russell H. Morgan Department of Radiology and Radiological Science, The Johns Hopkins University School of Medicine, Baltimore, MD, USA
Vladimir	Mlynarik	High‐field MR Centre, Department of Biomedical Imaging and Image‐guided Therapy, Medical University of Vienna, Vienna, Austria
Ewald	Moser	High‐field MR Centre, Centre for Medical Physics and Biomedical Engineering, Medical University of Vienna, Vienna, Austria.
Robert	Mulkern	Harvard Medical School, Boston, MA, USA and Department of Radiology, Boston Children's Hospital, Boston, MA, USA
Paul	Mullins	Bangor Imaging Center, School of Psychology, Bangor University, UK
Georg	Oeltzschner	Russell H. Morgan Department of Radiology and Radiological Science, Johns Hopkins University School of Medicine, USA
Ricardo	Otazo	New York University School of Medicine, New York, NY, USA
Esin	Ozturk‐Isik	Institute of Biomedical Engineering, Bogazici University, Istanbul, Turkey
Ulrich	Pilatus	Goethe‐University, Institute of Neuroradiology, Frankfurt am Main, Germany
Jeanine J.	Prompers	Department of Radiology, University Medical Center Utrecht, Utrecht, the Netherlands
Caroline L.	Rae	Neuroscience Research Australia, NSW, Australia; School of Medical Science, The University of New South Wales, NSW, Australia
Eva‐Maria	Ratai	Massachusetts General Hospital, A. A. Martinos Center for Biomedical Imaging, and Harvard Medical School, Charlestown, MA, USA
Jimin	Ren	Advanced Imaging Research Center, University of Texas Southwestern Medical Center, Dallas, USA
Chris	Rodgers	Wolfson Brain Imaging Centre, Department of Clinical Neurosciences, University of Cambridge, Cambridge, United Kingdom.
Tom W.J.	Scheenen	Radiology and Nuclear Medicine, Radboud University medical center, Nijmegen, the Netherlands
Albrecht	Schmid	High‐field MR Center, Centre for Medical Physics and Biomedical Engineering, Medical University of Vienna, Vienna, Austria.
Rolf F.	Schulte	GE Healthcare, Munich, Germany
Noam	Shemesh	Champalimaud Research, Champalimaud Centre for the Unknown, Lisbon, Portugal
Brian J.	Soher	Department of Radiology, Duke University Medical Center, Durham, North Carolina, USA
Bernhard	Strasser	Martinos Center for Biomedical Imaging, Department of Radiology, Massachusetts General Hospital, Harvard Medical School, Boston, MA, USA
Assaf	Tal	Department of Chemical and Biological Physics, Weizmann Institute of Science, Israel
Shang‐yueh	Tsai	Graduate Institute of Applied Physics, National Chengchi University, Taiwan
Julien	Valette	Commissariat à l'Energie Atomique et aux Energies Alternatives (CEA), Fontenay‐aux‐Roses, France; Neurodegenerative Diseases Laboratory, CEA, CNRS, Université Paris Sud, Université Paris‐Saclay, France
Ladislav	Valkovič	Oxford Centre for Clinical MR Research, University of Oxford, Oxford, UK
Marinette	van der Graaf	Department of Radiology and Nuclear Medicine, Radboud University Medical Center, Nijmegen, The Netherlands
Sabine	van Huffel	Stadius Center for Dynamical Systems, Signal Processing and Data Analytics, Department of Electrical Engineering (ESAT), KU Leuven, Leuven, Belgium
Jannie P.	Wijnen	University Medical Centre Utrecht, Utrecht, the Netherlands
Lijing	Xin	Centre d'Imagerie Biomedicale (CIBM), Ecole Polytechnique Fédérale de (EPFL), Lausanne, Switzerland
Xin	Yu	Department of Biomedical Engineering & Department of Radiology, Case Western Reserve University, Cleveland, OH, USA.
